# LINC02015 modulates the cell proliferation and apoptosis of aortic vascular smooth muscle cells by transcriptional regulation and protein interaction network

**DOI:** 10.1038/s41420-023-01601-z

**Published:** 2023-08-18

**Authors:** Fangyu Liu, Yulin Wang, Xitong Huang, Dingqian Liu, Wenjun Ding, Hao Lai, Chunsheng Wang, Qiang Ji

**Affiliations:** 1 Department of Cardiovascular Surgery, Zhongshan Hospital, Fudan University, Shanghai, 200032 China; 2 Shanghai Municipal Institute for Cardiovascular Diseases, Shanghai, 200032 China; 3 Department of Traditional Chinese Medicine, China Pharmaceutical University, Nanjing, Jiangsu 211198 China

**Keywords:** Apoptosis, Aortic diseases, Long non-coding RNAs, Protein-protein interaction networks, Transcriptional regulatory elements

## Abstract

Long intergenic nonprotein coding RNA 2015 (LINC02015) is a long non-coding RNA that has been found elevated in various cell proliferation-related diseases. However, the functions and interactive mechanism of LINC02015 remain unknown. This study aimed to explore the role of LINC02015 in the cell proliferation and apoptosis of vascular smooth muscle cells (VSMCs) to explain the pathogenesis of aortic diseases. Ascending aorta samples and angiotensin-II (AT-II) treated primary human aortic VSMCs (HAVSMCs) were used to evaluate the LINC02015 expression. RNA sequencing, chromatin isolation by RNA purification sequencing, RNA pull-down, and mass spectrometry (MS) were applied to explore the potential interacting mechanisms. LINC02015 expression was found elevated in aortic dissection and AT-II-treated HAVSMCs. Cell proliferation and cell cycle were activated in HAVSMCs with LINC02015 knockdown. The cyclins family and caspase family were found to participate in regulating the cell cycle and apoptosis via the NF-κB signaling pathway. RXRA was discovered as a possible hub gene for LINC02015 transcriptional regulating networks. Besides, the protein interaction network of LINC02015 was revealed with candidate regulating molecules. It was concluded that the knockdown of LINC02015 could promote cell proliferation and inhibit the apoptosis of HAVSMCs through an RXRA-related transcriptional regulation network, which could provide a potential therapeutic target for aortic diseases.

## Introduction

Long non-coding RNA (lncRNA) is a group of RNAs that are functionally active in multiple biological processes and participates in gene regulation through different pathways in cardiovascular diseases [1]. The relationship between lncRNA and aortic diseases has been extensively studied, with several risk indicators of aortic diseases discovered, while noncoding RNA could not be ignored [2–5]. Previous studies have identified lncRNAs that could serve as biomarkers or therapeutic targets for aortic dissection (AD), including LINC01278, Lnc-OIP5-AS1, H19, PVT1, CDKN2B-AS1, XIST, and PTENP1 [refs. 6–11]. However, the origin of aortic diseases remains unclear, with complex environmental and hereditary factors thought to play a role.

Long intergenic non-protein coding RNA 2015 (LINC02015) is a lncRNA that shows the potential in regulating cell behaviors. In public databases, single nucleotide polymorphism of LINC02015 was shown to have a relationship with venous thromboembolism, calcium level, and several proliferative diseases [12, 13]. Besides, it has been suggested that LINC02015 is possibly related to vascular injury, calcium ion regulation, and cell proliferation, which are important in a lot of cardiovascular diseases [14, 15]. What’s more, through online Gene Expression Omnibus (GEO) profiles, we find different expression levels of LINC02015 in cardiovascular relating situations, including tobacco use and hypoxia, which could damage our arteries [16, 17]. However, there is no clue whether LINC02015 affects the cell behaviors of VSMCs, which are crucial in the pathogenesis of various aortic diseases.

The molecular mechanism of VSMCs in the pathogenesis of aortic diseases has been studied by various techniques. VSMCs are important cellular components of the arterial wall, providing structural stability to the artery [18]. Cell cycle adjustment and apoptosis are proven to have effects on the proliferation and morphology of VSMCs [19, 20]. The altered cell function and morphology of VSMCs could influence the development of multiple cardiovascular diseases [21].

In previous studies, LINC02015 was thought to be related to multiple cell proliferation-related diseases. LINC02015 was initially thought to be a risk factor in postoperative glioblastoma [22]. With more investigations, LINC02015 was found to be differentially expressed in gastric cancer cells and metastatic esophageal squamous cell carcinoma [23, 24]. Besides, LINC02015 was also proved to be related to metabolic disorders in adipose tissue in type 2 diabetes individuals [25]. Recently, LINC02015 was reported to interact with miRNA-888 in malignant pleural mesothelioma [26]. The role of LINC02015 in SARS-CoV-2 infection also attracted researchers’ attention while potentially interacting with SARS-CoV-2 interacting host proteins UPF1 and MOV10 [ref. 27]. However, the interacting mechanism of LINC02015 remains to be validated, especially in the cardiovascular field.

In this study, we hypothesized that LINC02015 played an important role in regulating the cell proliferation and apoptosis of VSMCs. LINC02015 was elevated under pathological conditions of the aorta or angiotensin-II treatment. LINC02015 knockdown could promote the cell proliferation of VSMCs through various pathways. Besides, the inner interacting mechanism of LINC02015 with different molecular levels was investigated using mass spectrometry (MS) and next-generation sequencing (NGS). Our findings might provide new insights into the role of LINC02015 in the pathogenesis of aortic diseases and could have implications for the development of new therapeutic strategies.

## Results

### Expression of LINC02015 in aorta samples and AT-II-treated primary human aortic VSMCs (HAVSMCs)

**Fig. 1:**
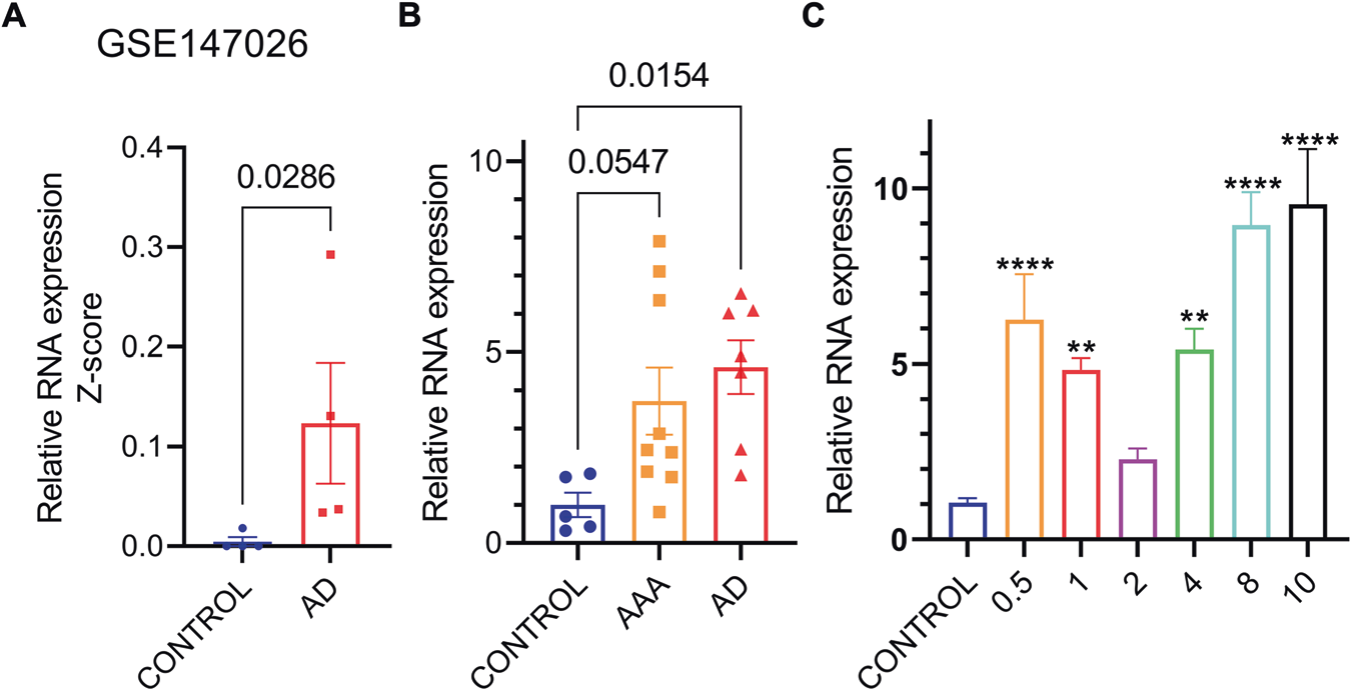
Expression of LINC02015 in aortic samples and angiotensin-II treated HAVSMCs. **A** LINC02015 expression of AD samples from GSE147026 (*N* = 4). **B** LINC02015 expression grouped by aortic lesion type (*N* = 5 for control, *N* = 9 for AAA, N = 7 for AD). **C** LINC02015 expression in HAVSMCs treated with 0.5/1/2/4/8/10 μM of AT-II for 24 h (*N* = 3). AAA, ascending aortic aneurysm; AD, aortic dissection. **p < 0.01, ****p < 0.0001. Data are presented as mean ± SEM.

We measured the LINC02015 expression in different conditions. In the GEO database, LINC02015 was shown to be elevated in AD samples of the dataset GSE147026 (Fig. 1A). The relative RNA expression of LINC02015 normalized to GAPDH was analyzed in 21 surgical aorta samples. The LINC02015 expression in the AD group was significantly higher than that in the control group with an average fold change of 4.606. Although no statistical difference was found between the ascending aortic aneurysm (AAA) and the control groups, there was a rising trend with an average fold change of 3.717 (Fig. 1B). Then we simulated this effect in vitro with AT-II treatment in primary HAVSMCs for 24 h. A concentration of 0.5/1/2/4/8/10 μM gradient was set and the LINC02015 expression was found to be elevated in all groups (Fig. 1C).

### Localization and knockdown of LINC02015

**Fig. 2:**
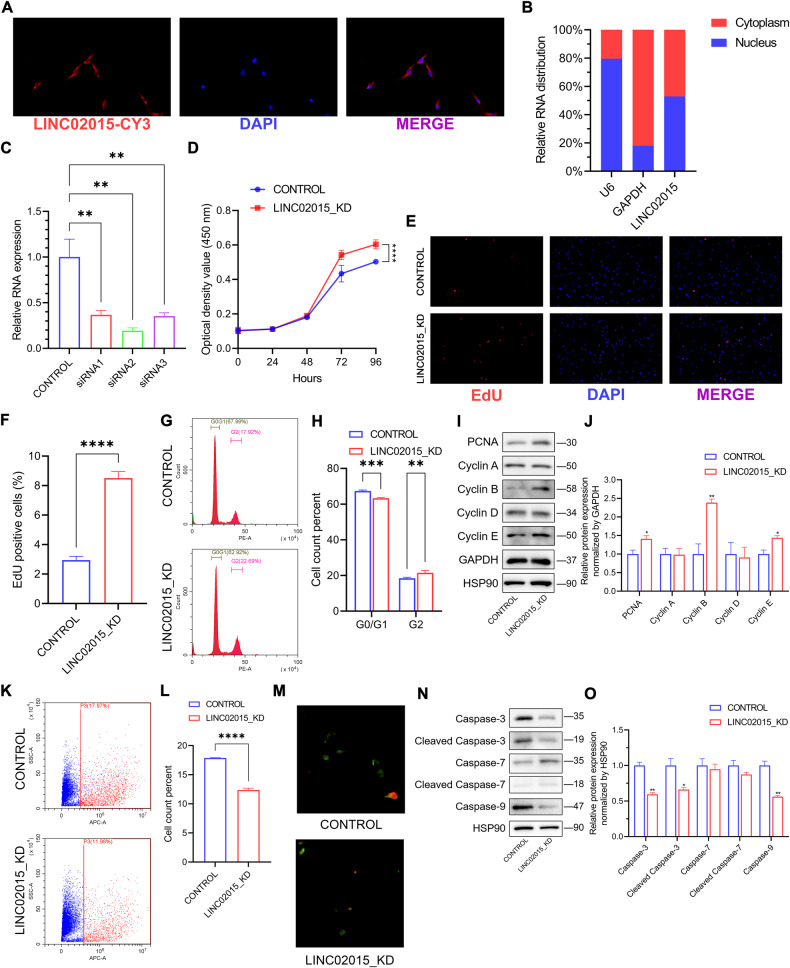
Distribution and biological function of LINC02015 in HAVSMCs. **A** FISH imaging for LINC02015 probes and nucleus with LINC02015 in red, DAPI in blue. **B** Intracellular distribution of LINC02015 quantified by qRT-PCR (*N* = 3). **C** LINC02015 knockdown efficiency quantified by qRT-PCR normalized by GAPDH (*N* = 3). **D** Cell viability measured by CCK-8 at 24, 48, 72 and 96 h (*N* = 6). **E** EdU assays showing proliferating cells stained by EdU in red, DAPI in blue. **F** The proportion of EdU-positive cells was calculated according to the total cell count in each field. (*N* = 4). **G, H** Cell cycle of control or LINC02015 knockdown cells presented by flow cytometry (*N* = 3). **I, J** Western blot analysis of PCNA and cyclins family under LINC02015 knockdown. Relative expression levels compared with control were normalized by GAPDH. **K, L** Apoptotic cells of control or LINC02015 knockdown cells presented by flow cytometry. (*N* = 3). **M** Annexin V/PI double staining of control or LINC02015 knockdown cells. Annexin V was shown in green and PI was shown in red. **N, O** Western blot analysis of apoptosis markers under LINC02015 knockdown. Relative expression levels compared with control were normalized by HSP90. FISH, fluorescence in situ hybridization. *p < 0.05, **p < 0.01, ***p < 0.001, ****p < 0.0001. Data are presented as mean ± SEM.

To manipulate LINC02015 expression, we detected the intracellular distribution of LINC02015 and then knocked down the LINC02015 with designed siRNAs. In HAVSMCs, we found that LINC02015 existed both in the nucleus and cytoplasm with fluorescence in situ hybridization (FISH) probes. We detected the expression of LINC02015 separately and found that LINC02015 was distributed almost evenly on both sides of the nuclear membrane (Fig. 2A, B). Then we used three designed siRNAs which were transfected into the cells and successfully knockdown the expression of LINC02015 (Fig. 2C).

### Knockdown of LINC02015 promotes cell proliferation and cell cycle and inhibits apoptosis

After transfecting the siRNA or negative control into the cells, cell viability, cell proliferation, cell cycle, and apoptosis of HAVSMCs were evaluated. It was shown that within 96 h since seeded, knockdown of LINC02015 could promote the cell viability of HAVSMCs reflected by CCK-8 (Fig. 2D). The proportion of proliferating cells was higher after LINC02015 knockdown with EdU assay (Fig. 2E, F). The proportion of G0/G1 cells was lower after the LINC02015 knockdown (Fig. 2G, H). PCNA, CCNB1, and CCNE1 were found elevated after knockdown (Fig. 2I, J). The apoptotic cells markedly decreased under the condition of LINC02015 knockdown (Fig. 2K-M). Thus, we detected apoptosis markers and found that total Caspase-3, cleaved Caspase-3, and total Caspase-9 were significantly reduced after the LINC02015 knockdown (Fig. 2N, O). Cleaved Caspase-9 was negative in both groups. LINC02015 may participate in modulating cell proliferation, cell cycle, and apoptosis by affecting the balance inside the cyclins family and caspase family.

### LINC02015 associates with multiple pathways through RNA regulation

**Fig. 3:**
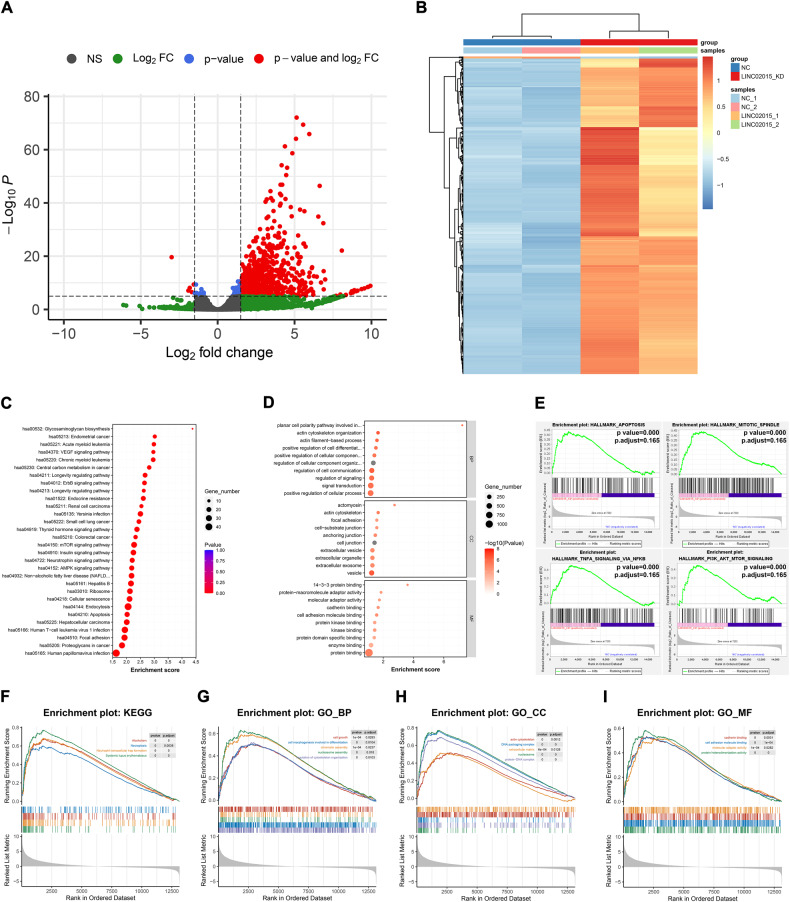
RNA sequencing and bioinformatic analysis of LINC02015 knockdown in HAVSMCs. **A, B** Volcano plot and heat map of differentially expressed genes between negative control and LINC02015 knockdown cells. **C, D** KEGG and GO functional enrichment analysis of up-regulated differentially expressed genes between control and LINC02015 knockdown cells. **E**–**I** GSEA analysis of expression profiles between control and LINC02015 knockdown cells in MSigDB, KEGG, and GO databases. BP biological process, CC cellular component, MF molecular function.

To explore the potential correlative RNAs with LINC02015, we used RNA sequencing (RNA-seq) to detect the variation of 14 916 candidate RNAs after the knockdown of LINC02015 (Table S1). The log2 fold change threshold was set at >2.0 or <-2.0 while the -log10*p*. adjust threshold was set at >3. 809 differentially expressed genes (DEGs) were filtered among the candidates (Fig. 3A, B, and Table S2).

Kyoto Encyclopedia for Genes and Genomes (KEGG) pathway analysis of up-regulated DEGs suggested that glycosaminoglycan biosynthesis may participate in the LINC02015 downstream regulation. Besides, several signaling pathways, such as VEGF or ErbB pathways were potentially involved. LINC02015 knockdown was also related to various kinds of proliferative diseases, including endometrial cancer and myeloid leukemia (Fig. 3C). Gene Ontology (GO) enrichment analysis of up-regulated DEGs indicated that LINC02015 may associate with planar cell polarity, actin cytoskeleton organization, and actin filament-based process. These biological processes could influence cell extension and morphology. According to the cellular components, we found that LINC02015 may link to actomyosin, actin cytoskeleton, and focal adhesion, which were important in the pathophysiological process of aortic diseases. For molecular function, 14-3-3 protein binding, protein-macromolecule adaptor activity, and molecular adaptor activity were the top 3 enriched terms (Fig. 3D).

To validate the DEG enrichment analysis results, Gene Set Enrichment Analysis (GSEA) analysis was also applied in Molecular Signatures Database (MSigDB), KEGG, and GO databases. For MSigDB Hallmark pathways, 15 pathways, including apoptosis, mitotic spindle, TNF-α signaling via NF-κB, and PI3K-AKT-mTOR signaling were enriched at *p* < 0.01 and *p*.adjust < 0.25 (Fig. 3E). For KEGG and GO analysis, we set the criterion at *p* < 0.001 and *p*.adjust < 0.05. Four KEGG pathways, including necroptosis were enriched (Fig. 3F). 41 biological processes were enriched, including terms related to cell growth and replication (Fig. 3G). 50 cell components were enriched with terms regarding DNA packaging, cytoskeleton, and extracellular matrix (Fig. 3H). Four molecular function terms were enriched, including cadherin binding, cell adhesion binding, molecular adaptor activity, and protein heterodimerization activity (Fig. 3I).

### Genome localization and functional enrichment of LINC02015 interacting genes

**Fig. 4:**
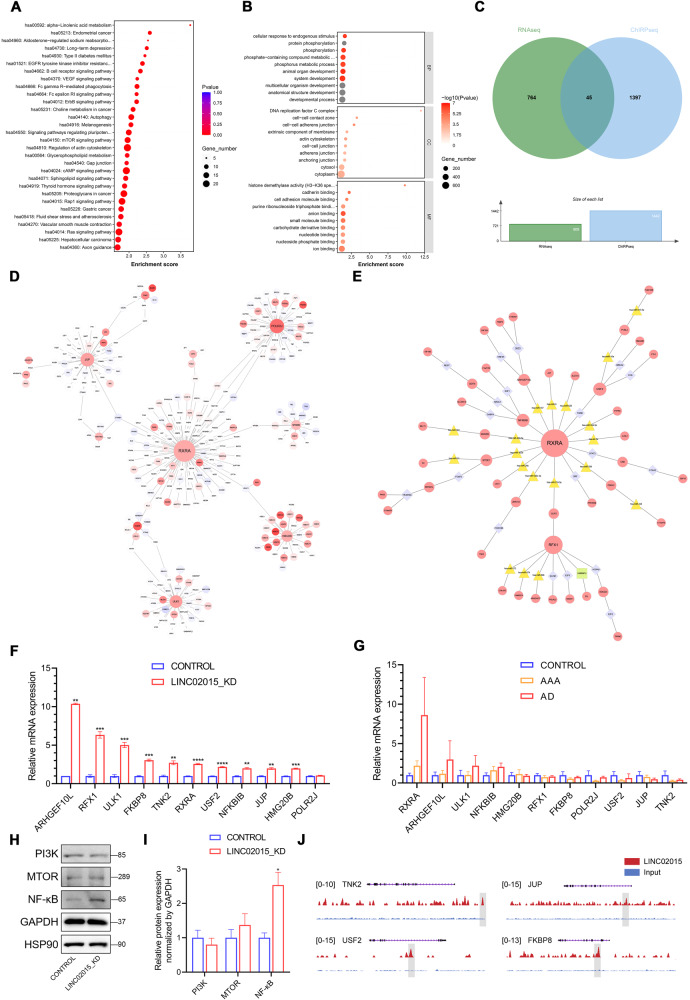
ChIRP sequencing and downstream genes of LINC02015 knockdown. **A, B** KEGG and GO enrichment analysis of identified gene peaks on the genome. **C** Venn diagram of DEGs and gene peaks; **D** PPI network of coregulatory genes. Blue to red stands for low expression to high, size of the node stands for interactive degrees. **E** TF-miRNA-Gene coregulatory network of coregulatory genes. Colors and shapes stand for molecular types, size of the node stands for interactive degrees. **F** mRNA expression levels of key node genes normalized to GAPDH in LINC02015 knockdown HAVSMCs (*N* = 3). **G** mRNA expression levels of key node genes normalized to GAPDH in aortic samples (*N* = 5 for control, *N* = 9 for AAA, *N* = 7 for AD). **H, I** Western blot analysis of downstream pathways under LINC02015 knockdown. Relative expression levels compared with Control were normalized by GAPDH. **J** Localization of LINC02015 binding sites of targeted genes. AAA ascending aortic aneurysm, AD aortic dissection. *p < 0.05, **p < 0.01, ***p < 0.001, ****p < 0.0001. Data are presented as mean ± SEM.

Through data cleaning and comparison, gene reads of Chromatin isolation by RNA purification sequencing (ChIRP-seq) could be identified. 1 695 gene peaks were scanned, clustered, and annotated (Table S3). GO and KEGG analyses were also applied to these gene peaks (Fig. 4A, B). Several terms relating to the cell cycle were also enriched, such as DNA replication factor C complex and histone demethylase activity. Motif analysis revealed 22 possible motifs located in the peak regions on the genome (Table S4). Then four known motifs were matched, including Gfi1b and GSC (Table S4).

### Transcriptional regulating network construction for LINC02015 interplaying genes

To explore the gene-regulating network of LINC02015, the DEGs from RNA-seq and gene peaks from ChIRP-seq were intersected. A total of 1442 genes from ChIRP-seq were taken into account after calculating the replicates. 45 genes were screened out, suggesting a potentially transcriptional-level regulated gene set (Fig. 4C). Then the protein-protein interaction (PPI) network was constructed within this gene set (Fig. 4D). We discovered that RXRA could potentially act as the hub gene in the LINC02015 regulating network. Furthermore, Transcription factors (TF)-MicroRNA (miRNA)-Gene coregulatory network was predicted with the 45 genes. Interacting TFs and miRNAs were arranged with the core genes to form a potential coregulatory network (Fig. 4E). Vital node genes were revealed from the networks, such as FKBP8, ULK1, HMG20B, RFX1, NFKBIB, POLR2J, USF2, JUP, TNK2, and ARHGEF10L. Then we validated these gene expressions in LINC02015 knockdown HAVSMCs by RT-qPCR. All these important node gene expressions were elevated except for POLR2J (Fig. 4F). Also, we intended to validate the expression difference in the aorta samples. TNK2, JUP, USF2, POLR2J, and FKBP8 showed decreased expressions in either pathological condition with a relatively high level of LINC02015 expression. However, RXRA, ARHGEF10L, ULK1, and NFKBIB showed unexpectedly higher expression in AD samples (Fig. 4G). Changes in enriched NF-κB and PI3K signaling pathways were also validated by Western blot. PI3K and mTOR showed differential expressions but with no significance, while NF-κB showed significantly higher expression after the LINC02015 knockdown (Fig. 4H, I). The LINC02015-binding sites of four targeted genes were exhibited (Fig. 4J). Our study revealed an RXRA-related LINC02015 downstream regulating network, which consisted of key genes and the NF-κB signaling pathway.

### LINC02015 interacts with multiple RNA-binding protein targets

**Fig. 5:**
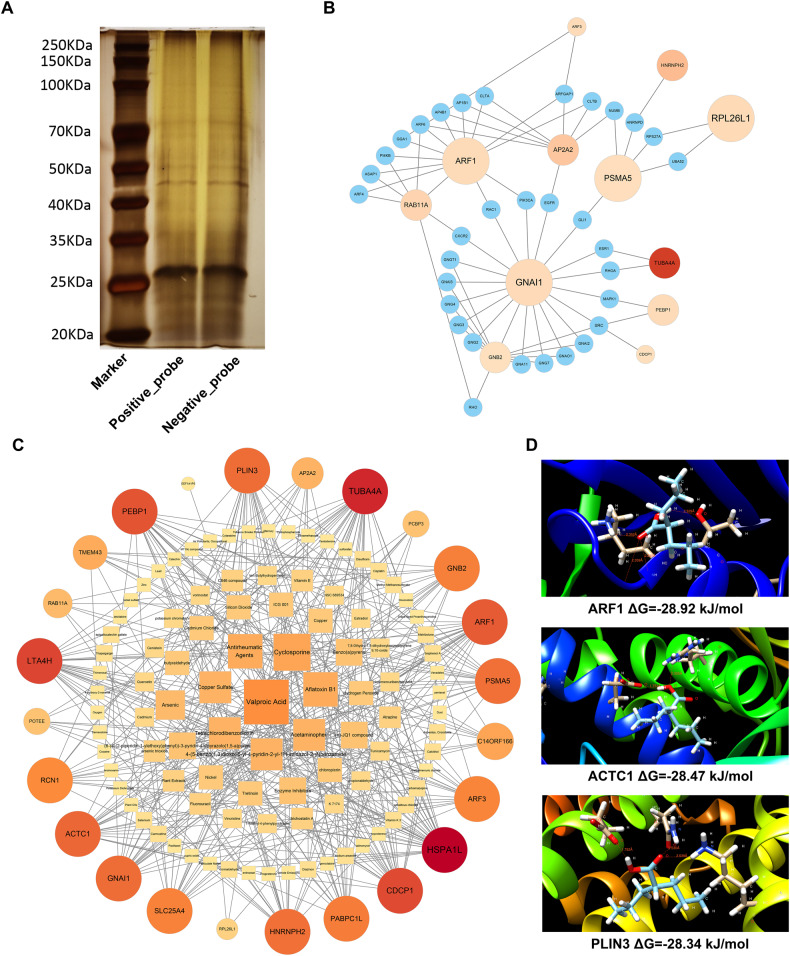
RNA pull-down products of LINC02015 and bioinformatic analysis of top 30 proteins. **A** Electrophoresis of the RNA pull-down products. **B** PPI network of top 30 LINC02015 binding proteins. **C** Potentially regulating chemical prediction with top 30 LINC02015 binding proteins. **D** Molecular docking models between valproic acid and ARF1, ACTC1, and PLIN3. The binding energy was listed below the picture.

Using MS, the RNA pull-down product was identified and analyzed (Fig. 5A). Respectively, 2 091 and 1 159 proteins were identified for the positive and negative probes. Through further comparison, 1 062 identified proteins were listed (Table S6). We applied GO and KEGG analysis to these proteins and listed the enriched pathways (Tables S7–S10). Notably, cellular component enrichment analysis suggested that extracellular exosome was associated with LINC02015 binding proteins. Then we input the top 30 LINC02015 binding proteins into PPI analysis. A PPI network was established with 12 LINC02015 binding proteins (Fig. 5B). To predict chemicals that share functional similarities with LINC02015, we screened the database to obtain small molecules that could interact with the top 30 LINC02015 binding proteins. Fourteen proteins out of the 30 proteins could interact with valproic acid (Fig. 5C). Then we predicted the optimal binding modes of valproic acid and these 14 proteins. All the proteins display potential binding ability. ARF1 showed the highest binding possibility with the estimated ΔG of -28.92 kJ/mol (-6.91 kcal/mol). Our data provided an additional regulating mechanism of LINC02015 at the protein-binding level.

## Discussion

Through this study, we validated the high expression of LINC02015 in aortic diseases and AT-II-treated HAVSMCs. LINC02015 knockdown could promote cell proliferation, activate the cell cycle, and inhibit apoptosis of HAVSMCs through an RXRA-related network. Manipulation of LINC02015 could serve as a new therapeutic candidate for aortic diseases. Our study put forward a new perspective between lncRNAs and aortic diseases. In recent years, non-coding RNAs became a hot spot in terms of gene regulation and intracellular signaling [28]. LncRNA appeared to be a kind of potential molecule that could perform as biomarkers or gene-regulating targets. Several classic lncRNAs took effect in disease diagnosis while clinical trials towards lncRNA targeting therapy are also in process [29]. There were still many mysteries about this small compound. With the developing sequencing techniques, more and more lncRNAs were found and annotated [30]. However, only a few of them were studied in limited disease models. The functioning mechanism and interacting characters remained to be figured out.

From validated views of previous studies, several important pathogenic factors were thought to contribute a lot to cardiovascular progression. For example, gene mutation causing systemic diseases such as Marfan syndrome (FBN1), Ehlers-Danlos syndrome (COL3A1), and Loeys-Dietz syndrome (TGFBR1/2) could significantly increase the risk of aortic dissection [31]. Vascular inflammation induced by infection or autoimmune diseases could also damage the stable arterial wall structure through various mechanisms, including matrix metalloproteinases (MMPs) and vascular endothelial growth factor (VEGF) related pathways [19]. Though lncRNAs did not usually encode proteins, their specific structural domains enabled their combining abilities with different molecules.

The current study demonstrated that LINC02015 expression was elevated in AD samples as well as AT-II-treated primary HAVSMCs. We validated the relationship between LINC02015 expression with aortic diseases and the pressure-induced stress model in vitro. AT-II was usually used to introduce cardiovascular disease models, including aortic aneurysm, aortic dissection, and myocardial hypertrophy [32]. The results suggested that LINC02015 could potentially act as an important mediator between the pressure response and pathological changes of multiple cardiovascular diseases.

Then we intended to reveal the cell behaviors of HAVSMCs with LINC02015 regulation. The distribution of LINC02015 was found to be almost balanced between the nucleus and cytoplasm, which indicated that LINC02015 might act as an intracellular signaling messenger across the nuclear membrane or take biological effects both in the nucleus and the cytoplasm. Knockdown of LINC02015 was feasible in HAVSMCs using siRNAs. LINC02015 knockdown could promote the cell proliferation of HAVSMCs by adjusting the cell cycle and inhibiting apoptosis. PCNA expression and EdU-positive cells were higher with LINC02015 knockdown, indicating a higher level of cell proliferation. In the cyclins family, we found that elevated cyclin B and cyclin E might take the main responsibility to activate the cell cycle after the LINC02015 knockdown. Cyclins family was also proven to take important effects on various kinds of cardiovascular diseases [33, 34]. But the relationship between cyclin B and aortic diseases was rarely reported. Another important finding was, the change in caspase subtypes could be the underlying LINC02015 downstream targets. Caspase-3 and Caspase-9 pathways were inhibited, resulting in a reduced apoptotic cell proportion. The fact indicates that LINC02015 might serve as a potential regulating target in the management of aortic disease progression by influencing cell growth and death in its unique way.

Later on, interacting mechanisms of LINC02015 in different molecular levels were investigated through several high-throughput analyses. In DEGs functional enrichment analysis and GSEA, we found some focused terms and pathways as follows. Glycosaminoglycan biosynthesis and extracellular vesicle/organelle/exosome/matrix were noted suggesting a close relationship between LINC02015 with extracellular matrix components [35]. The contribution of LINC02015 to extracellular communication deserved to be addressed in future studies. Some classical signaling pathways, such as the VEGF pathway, ErbB pathway, NF-κB pathway, and PI3K-AKT-mTOR pathway were enriched along with a lot of terms that participated in cell cycle, apoptosis, cytoskeleton organization, and cell adhesion. The impacts of LINC02015 knockdown on the HAVSMCs might not solely depend on a simple linear pathway but a complex interacting network.

As for the genome binding possibility of LINC02015, potential targets were discovered by peak calling analyses. Several terms related to the cell cycle were focused such as DNA replication factor C complex and histone demethylase activity, which might regulate important biological processes such as cell proliferation, aging, and tumorigenesis [36]. Besides, alpha−linolenic acid metabolism was enriched, which was consistent with a former study of metabolic disorders [25]. Motif analysis exhibited various binding motifs. Gfi1b and GSC were matched when we compared predicted motifs with known motifs. A possible regulating network among these pathways and interacting mediators was worth exploring with more effort.

PPI network and TF-miRNA-Gene coregulatory network were established to show an interacting network with a hub gene RXRA. RXRA and other important node genes, including BCL2, FKBP8, and NFKBIB also belonged to a sort of pathways that were related to cell survival and proliferating regulation [37–39]. In the PI3K-AKT-mTOR pathway, AKT could regulate cell survival by inhibiting BCL-2 via phosphorylated inhibition of RXRA. Besides, mi-26b, which was confirmed as a protective gene in cardiovascular diseases [40], was found to connect RXRA with ULK1 in the coregulatory network. ULK1 was also a protein-coding gene involved in autophagy. The validation results suggested a complex regulating mechanism among these RXRA-related genes. Higher expression of NF-κB induced by LINC02015 knockdown might be a key pathway during the regulating process. The NF-κB pathway was also related to VSMC phenotypes, which were important in vascular diseases [41, 42].

Meanwhile, in the MS enrichment analysis of LINC02015 binding proteins, extracellular exosome was speculated to be associated with LINC02015 interaction. In the PPI network, distinct LINC02015 binding proteins were centralized as important nodes. GNAI1 along with other G proteins played an important role in signal transduction. Other highly centralized proteins were involved in protein translation and intracellular traffic. To seek functionally similar chemicals with LINC02015, an online database was explored to rank the interacting level of the top 30 LINC02015 binding proteins. Valproic acid was screened out to have the highest potential to interact with the LINC02015 binding proteins. Valproic acid was also reported as a histone deacetylases inhibitor, which could reverse AT-II-induced cardiac hypertrophy, modulate the cell cycle and increase nitric oxide production of vascular endothelial cells [43–45]. The biological functions of valproic acid were highly associated with our results. This could provide us clues in explaining the mechanism of aortic disease progression from a new perspective.

Our study initially explored the relationship between LINC02015 expression and aortic diseases. Cell behaviors of HAVSMCs were observed under LINC02015 knockdown. We attempted to explain the interacting mechanism of LINC02015 from different molecular levels using several high throughput techniques. Possible interacting sites were revealed whether inside or outside the nucleus to provide candidate research direction. Interacting networks were constructed to primarily explain how this lncRNA works. However, limitations existed in our study. First, we only validated our findings at the cellular level. The findings were based on the functional changes in HAVSMCs. Second, apart from cell proliferation, other factors which also contributed to disease progressions, such as inflammation and stress remained to be studied. Finally, more work in the regulating mechanisms was needed for the fact that the lncRNA could influence biological behaviors in different ways.

## Materials And Methods

### Sample collection

All the aorta samples were obtained from patients who underwent cardiovascular surgery at Zhongshan Hospital Fudan University. The patient samples were collected sequentially in November 2021. The study protocol was proven by the ethics committee of Zhongshan Hospital Fudan University. Written consents were obtained on admission from the patients and/or their legal representatives. Three patients without aortic diseases received coronary artery bypass grafting where the aorta would be punched with a small hole, thus leaving a control sample. The other patients were diagnosed with AAA or AD requiring a replacement of the aorta. The study protocol was consistent with the *Declaration of Helsinki*.

### Cell culture

Primary HAVSMCs were isolated from donor aorta samples resected during surgery. The samples were cut into pieces and digested with collagenase-II overnight. The mixture was filtrated and centrifuged to separate suspending cells. Primary HAVSMCs were cultured with Smooth Muscle Cell Medium (#1101, Sciencell, USA). HAVSMC cell line was purchased from American Type Culture Collection (CRL-1999, ATCC, USA) and cultured with Ham’s F-12K medium (Gibco, NY, USA) containing 10% fetal bovine serum (Gibco, Australia) and other supplements suggested by ATCC. Cells were cultured in a humidified 37 °C incubator with 5% CO2. Cell passaging was executed when they became 80–90% confluent with 0.25% trypsin (Gibco, NY, USA) for 1 min and were collected, centrifuged, resuspended, and seeded at a 1:3 ratio. The cells were plated in 6-well plates and treated with different agents when they became 70–80% confluent. Primary HAVSMCs were only used to validate LINC02015 expression under different concentrations of AT-II for 24 h, which is to simulate the pressure-related stress in vitro. The other cell experiments were conducted with HAVSMC cell lines.

### Cell transfection

To knock down LINC02015, three small interfering RNAs (siRNAs) and a negative control siRNA were designed and constructed by Tsingke Biotechnology Co., Ltd. (Beijing, China). They were transfected into VSMCs with Lipofectamine RNAiMAX (Invitrogen, USA). All the procedures were under the manufacturer’s recommendation. The transfection medium was replaced after 24 h. All transfected VSMCs were collected after 48 h. The efficiency of transfection was determined by quantitative real-time polymerase chain reaction (qRT-PCR). The siRNA with the highest knockdown efficiency would be used in the following functional assays.

### QRT-PCR

LINC02015 knockdown efficiency and intracellular distribution were validated using qRT-PCR. Total RNA was extracted from cells using TRIzol Reagent (Invitrogen, Carlsbad, USA) for the measurement of LINC02015 knockdown efficiency. Cytoplasmic and nuclear RNA separation was executed with the Cytoplasmic and Nuclear RNA Purification Kit (Norgen Biotek, Canada). RNA samples were reversely transcribed to the cDNA library using the SuperScript III Reverse Transcriptase kit (Invitrogen, Carlsbad, USA). All the procedures were performed under the manufacturer’s recommendation. RNA expression was normalized to GAPDH. Relative expression was calculated using the 2^−ΔΔCt^ method. At least three repeated tests were carried out on each sample. Three biological replicates were set for each group.

### Fluorescence in situ hybridization

FISH was conducted using a GenePharma fluorescence in situ hybridization kit in accordance with the manufacturer’s directions. In brief, the cells were seeded and fixed with 4% paraformaldehyde and treated with 0.5% Triton in PBS followed by pre-hybridization. The fixed cells were then hybridized at 1 mM probe concentration overnight. LINC02015 FISH probes were designed and synthesized by GenePharma.

### Cell viability

The effects of LINC02015 on cell viability were explored by Cell Counting Kit-8 (Donjindo, Japan). The cells were counted and seeded into a 96-well plate with 5 000 cells per well. Then the cells were cultured for 24, and 48 h at 37 °C with 5% CO2. Then 10 μl CCK-8 reagent was added to each well and incubated for 1 h. The 450 nm absorbance was detected using an enzyme-labeling instrument. The optical density of each group was normalized with the standard control group.

### EdU assay

To detect the proportion of proliferating HAVSMCs, we performed EdU assays. Briefly, cells were seeded in 6-well plates and transfected with different siRNAs. At 24 h after transfection, cells were collected and reseeded for EdU assays. The EdU assay kit (Beyotime, China) was used according to the manufacturer’s instructions.

### Flow cytometry

Cell cycle and apoptosis were analyzed by flow cytometry when the VSMCs were collected and transferred to a Falcon tube. Cells were collected and rinsed before staining with a Cell cycle and apoptosis analysis kit (Beyotime, China) and Annexin V-Alexa Fluor 647 (Yeasen, China). The mixture was loaded onto the flow cytometer after 15 min reaction in the darkness. Data were analyzed for cell cycle and apoptosis with Cell Quest software (BD Biosciences). The Annexin V/PI dual staining cells were also observed with a fluorescence microscope to evaluate the level of apoptosis.

### Western blot

Cells were lysed using RIPA buffer (Beyotime, China) containing protease and phosphatase inhibitors. Cell lysates were centrifuged at 10,000 rpm for 10 min and then the supernatants were separated. 5X loading buffer was mixed and heated at 95 °C. Total proteins were separated using sodium dodecyl sulfate-polyacrylamide gel electrophoresis (SDS-PAGE) and transferred onto polyvinylidene fluoride (PVDF) membranes. Incubation with antibodies was performed at 4 °C overnight after blocking the membrane for 60 min. The quantification was performed in three biological replicates and measured by relative expression levels of integrated optical density normalized by the loading control.

### RNA-seq

Total RNA was extracted from cells using TRIzol Reagent (Invitrogen, Carlsbad, USA). RNA concentration and purity were measured by NanoDrop 2000 Spectrophotometer (Thermo Fisher Scientific, Wilmington, DE). A total amount of 1.5 μg RNA per sample was reversely transcribed to the cDNA library using NEBNext Ultra Directional RNA Library Prep Kit for Illumina (NEB, USA) following the manufacturer’s recommendations. The library preparations were sequenced on an Illumina Hiseq platform and paired-end reads were generated. Raw data were analyzed with the DESeq2 R package after data cleaning, normalization, and annotation. The DEGs were statistically significant with a fold change of >2.0 or <-2.0 with -log10*p*.adjust >3. The original RNA-seq dataset could be retrieved from the GEO database as GSE188964.

### ChIRP sequencing

ChIRP-seq was applied to explore the interactive relationship between lncRNA and potential binding sites on chromosomes. Localization of the active binding sites was specified by high-throughput sequencing. The sequencing data were compared with the reference genome using STAR2 software. No more than 2 base mismatches were allowed and only reads related to a unique location on the genome would be used for subsequent analysis. Peak calling analysis was applied to spot enriched gene peaks. The localization of gene peaks was visualized by IGV software. MACS software was used for discovering differentially enriched gene peaks and Homer software was used for gene annotation and motif enrichment analysis. The original ChIRP-seq dataset could be retrieved from the GEO database as GSE188751.

### RNA pull-down

Potential interaction between LINC02015 and targeted binding proteins was analyzed by RNA pull-down assay. Cells were fixed and ultrasonically lysed before RNA pull-down. Biotin-labeled probes were synthesized according to the LINC02015 sequence. Cell samples and protein-RNA hybridization buffer were mixed in a 1:2 ratio before adding the probes. Magnetic beads were used to collect the RNA-protein binding product. The product was separated by gel electrophoresis and examined by protein MS analysis.

### Bioinformatic analysis

The sequencing results and MS results were analyzed using R version 4.1.2 with R pack-ages including ‘ggplot2’, ‘clusterProlifer’, ‘EnhancedVolcano’, and ‘pheatmap’. KEGG, GO, and MSigDB databases were used for gene enrichment analysis. Signaling pathways and biological terms were enriched by R version 4.1.2 or GSEA. PPI networks were constructed both in RNAseq-ChIRPseq coregulatory genes and RNA pull-down proteins with the STRING database. The TF-miRNA-Gene coregulatory network was explored with RegNetwork Repository. Possible chemicals sharing similar protein-interacting abilities with LINC02015 were discovered in the Comparative Toxicogenomics Database. Interacting networks of LINC02015 were visualized by Cytoscape version 3.9.1. Protein structures were downloaded from the Protein Data Bank or AlphaFold databases. Molecular docking was performed with SwissDock to predict the best binding site and was visualized by UCSF Chimera version 1.1.6.

### Statistical analysis

Experimental data were presented as mean ± SEM and analyzed using SPSS 26.0 (SPSS Inc., Chicago, IL). Differences between groups were compared by Student’s *t*-test or one-way ANOVA. The difference was considered statistically significant at a *p* value < 0.05.

### Supplementary information

Supplementary tables legends

Supplementary table S1_S3 S6_S10

Supplementary table S4_S5

Original Image for blots

## Data Availability

The original sequencing data used to support the findings of this study have been deposited in the GEO repository (GSE188964 and GSE188751). The processed sequencing data and RNA pull-down mass spectrometry data used to support the findings of this study are included in the supplementary files.
