# Supplementary material for: LINC02015 modulates the cell proliferation and apoptosis of aortic vascular smooth muscle cells by transcriptional regulation and protein interaction network
Source: Cell Death Discov. 2023 Aug 18;9:301. doi: 10.1038/s41420-023-01601-z (PMC10439127; doi:10.1038/s41420-023-01601-z)
Supplement: Supplementary file 1 — Supplementary tables legends [file 41420_2023_1601_MOESM1_ESM.docx]

**Supplementary tables legends**

Table S1: RNA sequencing details of 14916 candidate genes with LINC02015 knockdown

Table S2: Filtered details of 809 differentially expressed genes with LINC02015 knockdown

Table S3: LINC02015 peak calling details of 1695 gene peaks with ChIRP sequencing

Table S4: Homer motifs derived from ChIRP sequencing results

Table S5: Enriched known motifs derived from ChIRP sequencing results

Table S6: Details of 1062 identified proteins by RNA pull-down with LINC02015 probes

Table S7: Top 20 GO enriched terms of RNA pull-down proteins in the biological process

Table S8: Top 20 GO enriched terms of RNA pull-down proteins in cellular component

Table S9: Top 20 GO enriched terms of RNA pull-down proteins in molecular function

Table S10: Top 20 KEGG enriched terms of RNA pull-down proteins
