# Supplementary material for: LINC02015 modulates the cell proliferation and apoptosis of aortic vascular smooth muscle cells by transcriptional regulation and protein interaction network
Source: Cell Death Discov. 2023 Aug 18;9:301. doi: 10.1038/s41420-023-01601-z (PMC10439127; doi:10.1038/s41420-023-01601-z)
Supplement: Supplementary file 3 — Supplementary table S4_S5 [file 41420_2023_1601_MOESM3_ESM.docx]

**Table S4: Homer motifs derived from ChIRP sequencing results**

| **Rank** | **Motif** | **p value** | **Log p value** | **% of targets** | **% of background** | **STD(Bg STD)** | **Best match/Details** |
| --- | --- | --- | --- | --- | --- | --- | --- |
| 1 | 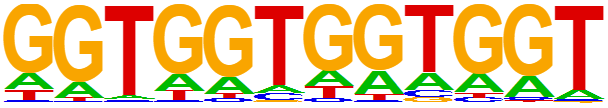 | 1e-128 | -2.955e+02 | 7.08% | 0.25% | 54.9bp (112.3bp) | ZBTB7C/MA0695.1/Jaspar(0.661) |
| 2 | 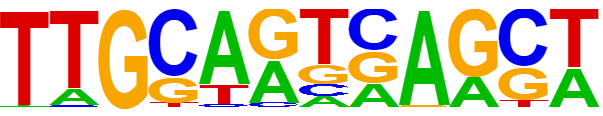 | 1e-118 | -2.720e+02 | 15.04% | 2.40% | 64.8bp (73.4bp) | PB0145.1_Mafb_2/Jaspar(0.611) |
| 3 | 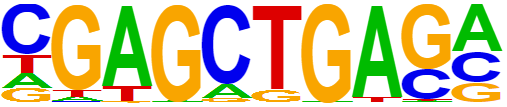 | 1e-108 | -2.504e+02 | 14.16% | 2.31% | 62.9bp (73.5bp) | NRL/MA0842.1/Jaspar(0.704) |
| 4 | 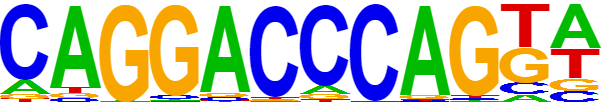 | 1e-103 | -2.388e+02 | 7.37% | 0.45% | 59.2bp (73.2bp) | LRF(Zf)/Erythroblasts-ZBTB7A-ChIP-Seq(GSE74977)/Homer(0.615) |
| 5 | 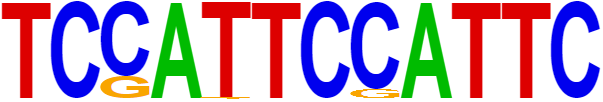 | 1e-102 | -2.357e+02 | 3.01% | 0.01% | 54.5bp (21.9bp) | PB0098.1_Zfp410_1/Jaspar(0.691) |
| 6 | 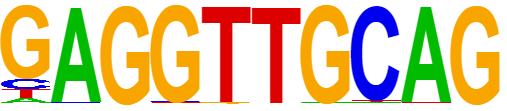 | 1e-65 | -1.518e+02 | 18.23% | 6.05% | 67.6bp (75.1bp) | SIX2/MA1119.1/Jaspar(0.674) |
| 7 | 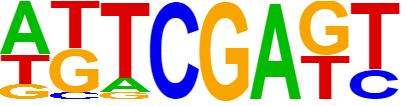 | 1e-41 | -9.643e+01 | 3.19% | 0.23% | 55.9bp (57.4bp) | CUX2/MA0755.1/Jaspar(0.645) |
| 8 | 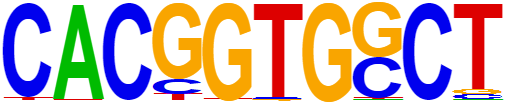 | 1e-28 | -6.574e+01 | 10.32% | 3.99% | 59.7bp (73.3bp) | PAX5(Paired,Homeobox),condensed/GM12878-PAX5-ChIP-Seq(GSE32465)/Homer(0.624) |
| 9 | 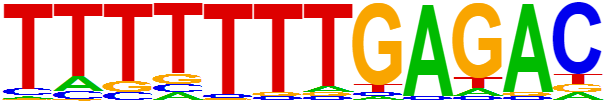 | 1e-23 | -5.517e+01 | 10.38% | 4.43% | 51.1bp (75.0bp) | ZNF384/MA1125.1/Jaspar(0.760) |
| 10 | 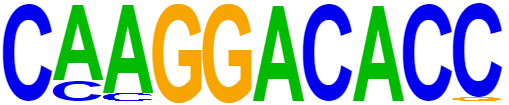 | 1e-21 | -4.853e+01 | 1.71% | 0.14% | 60.0bp (70.8bp) | SF1(NR)/H295R-Nr5a1-ChIP-Seq(GSE44220)/Homer(0.801) |
| 11 * | 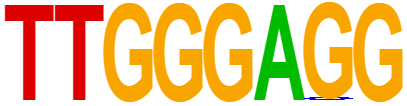 | 1e-10 | -2.394e+01 | 20.29% | 14.45% | 62.7bp (69.1bp) | PB0167.1_Sox13_2/Jaspar(0.722) |
| 12 * | 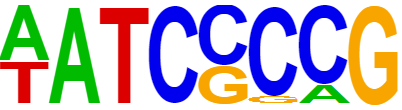 | 1e-10 | -2.343e+01 | 21.00% | 15.13% | 60.5bp (70.1bp) | MZF1/MA0056.1/Jaspar(0.741) |
| 13 * | 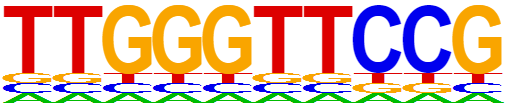 | 1e-9 | -2.095e+01 | 0.29% | 0.00% | 44.3bp (30.5bp) | IRF5/MA1420.1/Jaspar(0.670) |
| 14 * | 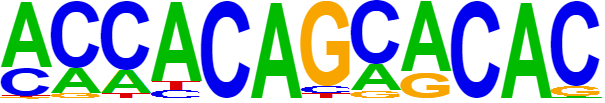 | 1e-8 | -2.050e+01 | 0.94% | 0.13% | 50.7bp (102.1bp) | RUNX-AML(Runt)/CD4+-PolII-ChIP-Seq(Barski_et_al.)/Homer(0.695) |
| 15 * | 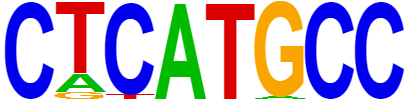 | 1e-6 | -1.605e+01 | 6.55% | 3.87% | 60.6bp (73.6bp) | HIC2/MA0738.1/Jaspar(0.701) |
| 16 * | 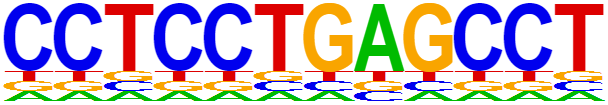 | 1e-3 | -8.259e+00 | 0.18% | 0.01% | 49.3bp (34.5bp) | Zic(Zf)/Cerebellum-ZIC1.2-ChIP-Seq(GSE60731)/Homer(0.657) |
| 17 * | 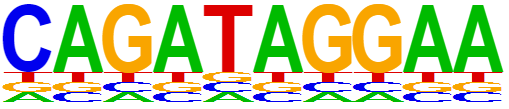 | 1e-3 | -7.573e+00 | 0.24% | 0.02% | 59.6bp (46.6bp) | Gata1/MA0035.3/Jaspar(0.745) |
| 18 * | 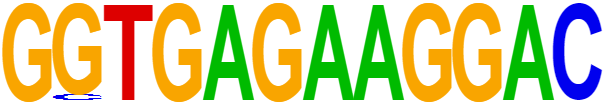 | 1e-2 | -6.786e+00 | 0.18% | 0.01% | 47.4bp (25.4bp) | TBX20/MA0689.1/Jaspar(0.648) |
| 19 * | 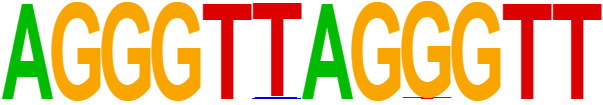 | 1e-2 | -5.835e+00 | 0.18% | 0.02% | 54.8bp (55.4bp) | ZNF652/HepG2-ZNF652.Flag-ChIP-Seq(Encode)/Homer(0.610) |
| 20 * | 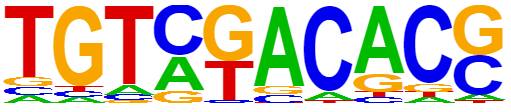 | 1e-1 | -2.982e+00 | 0.12% | 0.02% | 49.7bp (39.0bp) | Tbx20(T-box)/Heart-Tbx20-ChIP-Seq(GSE29636)/Homer(0.679) |
| 21 * | 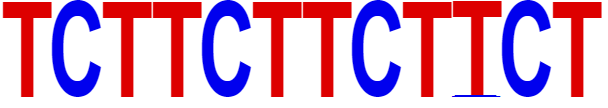 | 1e-1 | -2.632e+00 | 0.12% | 0.03% | 49.8bp (32.4bp) | SPIC/MA0687.1/Jaspar(0.584) |
| 22 * | 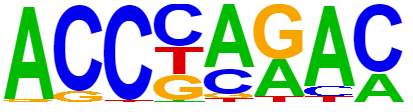 | 1e0 | -1.191e+00 | 1.30% | 1.14% | 53.5bp (82.7bp) | PB0035.1_Irf5_1/Jaspar(0.703) |

* possible false positive

**Table S5: Enriched known motifs derived from ChIRP sequencing results**

| **Rank** | **Motif** | **Name** | **p value** | **Log p value** | **q-value (Benjamini)** | **# Target Sequences with Motif** | **% of Targets Sequences with Motif** | **# Background Sequences with Motif** | **% of Background Sequences with Motif** |
| --- | --- | --- | --- | --- | --- | --- | --- | --- | --- |
| 1 | 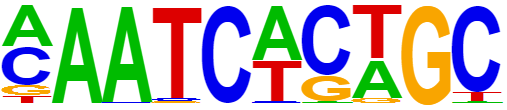 | Gfi1b(Zf)/HPC7-Gfi1b-ChIP-Seq(GSE22178)/Homer | 1e-9 | -2.098e+01 | 0.0000 | 244.0 | 14.40% | 4153.9 | 9.75% |
| 2 | 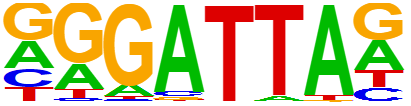 | GSC(Homeobox)/FrogEmbryos-GSC-ChIP-Seq(DRA000576)/Homer | 1e-6 | -1.452e+01 | 0.0001 | 512.0 | 30.21% | 10619.2 | 24.93% |
| 3 | 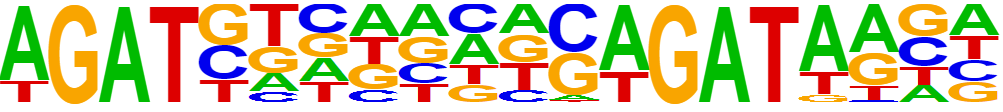 | GATA3(Zf),DR8/iTreg-Gata3-ChIP-Seq(GSE20898)/Homer | 1e-2 | -5.290e+00 | 0.7190 | 17.0 | 1.00% | 208.2 | 0.49% |
| 4 | 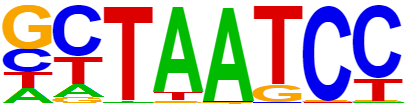 | CRX(Homeobox)/Retina-Crx-ChIP-Seq(GSE20012)/Homer | 1e-2 | -5.043e+00 | 0.7190 | 595.0 | 35.10% | 13731.4 | 32.23% |
