# Supplementary material for: LINC02015 modulates the cell proliferation and apoptosis of aortic vascular smooth muscle cells by transcriptional regulation and protein interaction network
Source: Cell Death Discov. 2023 Aug 18;9:301. doi: 10.1038/s41420-023-01601-z (PMC10439127; doi:10.1038/s41420-023-01601-z)

Original Image for Figure 2I_PCNA

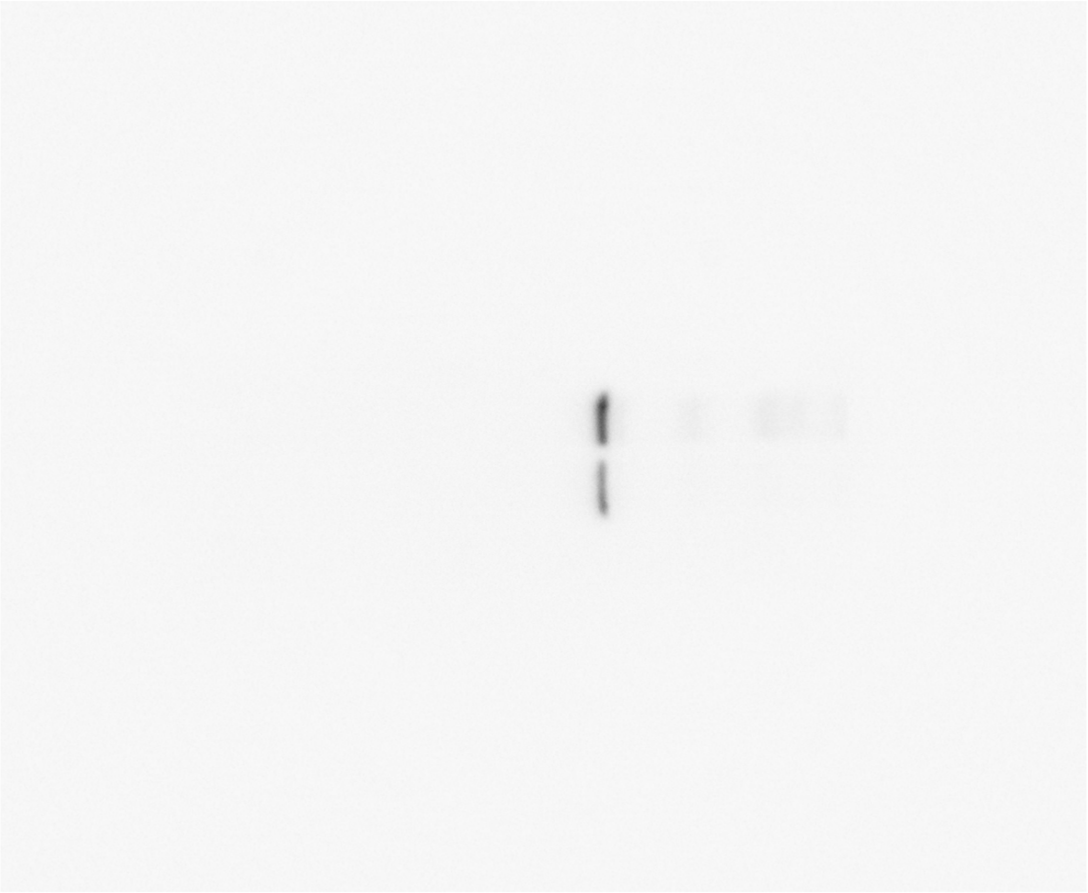

Original Image for Figure 2I_CCNA

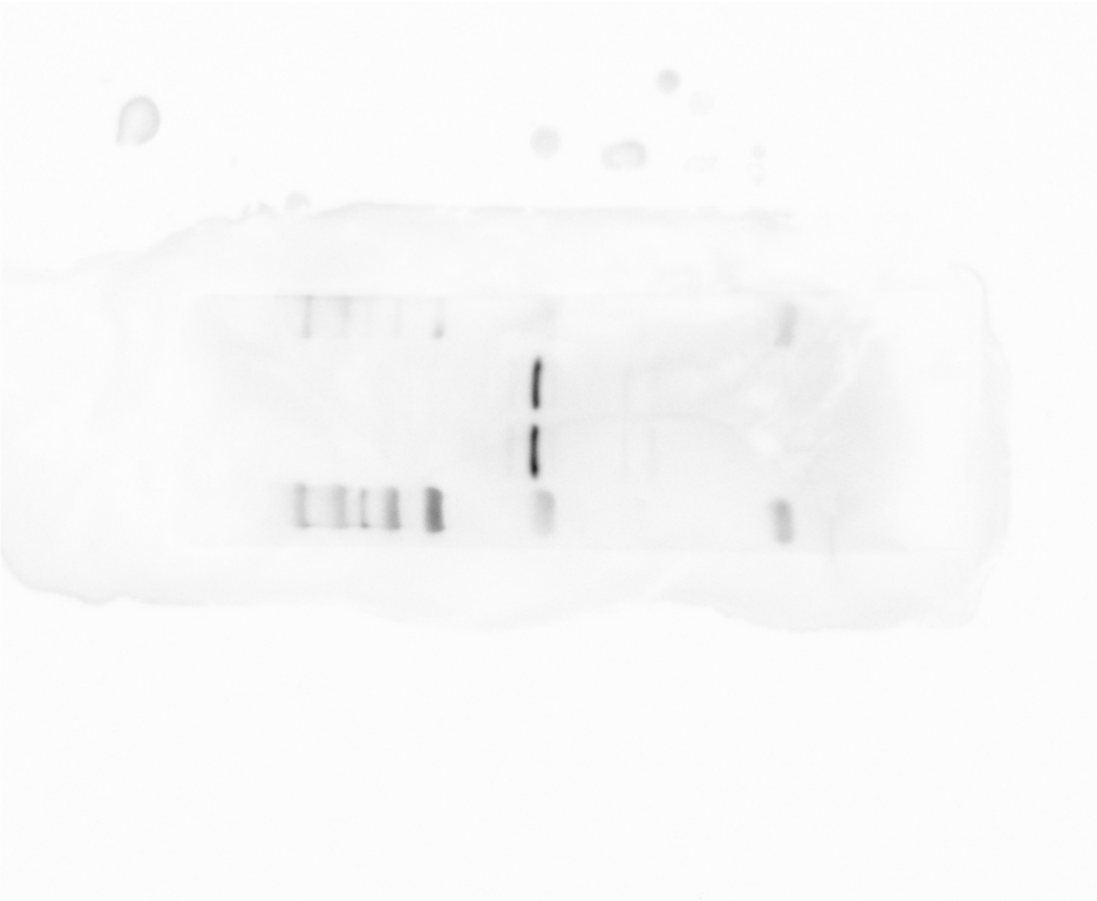

Original Image for Figure 2I_CCNB

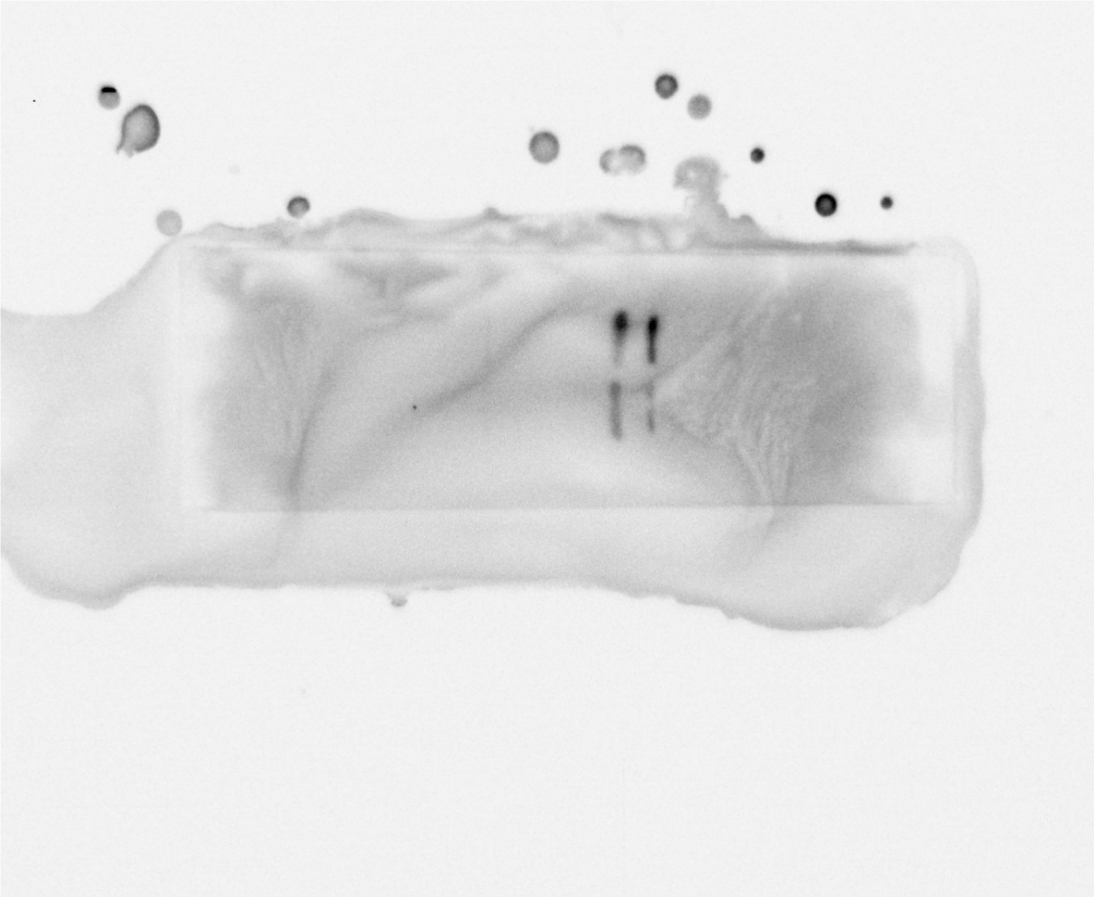

Original Image for Figure 2I_CCND

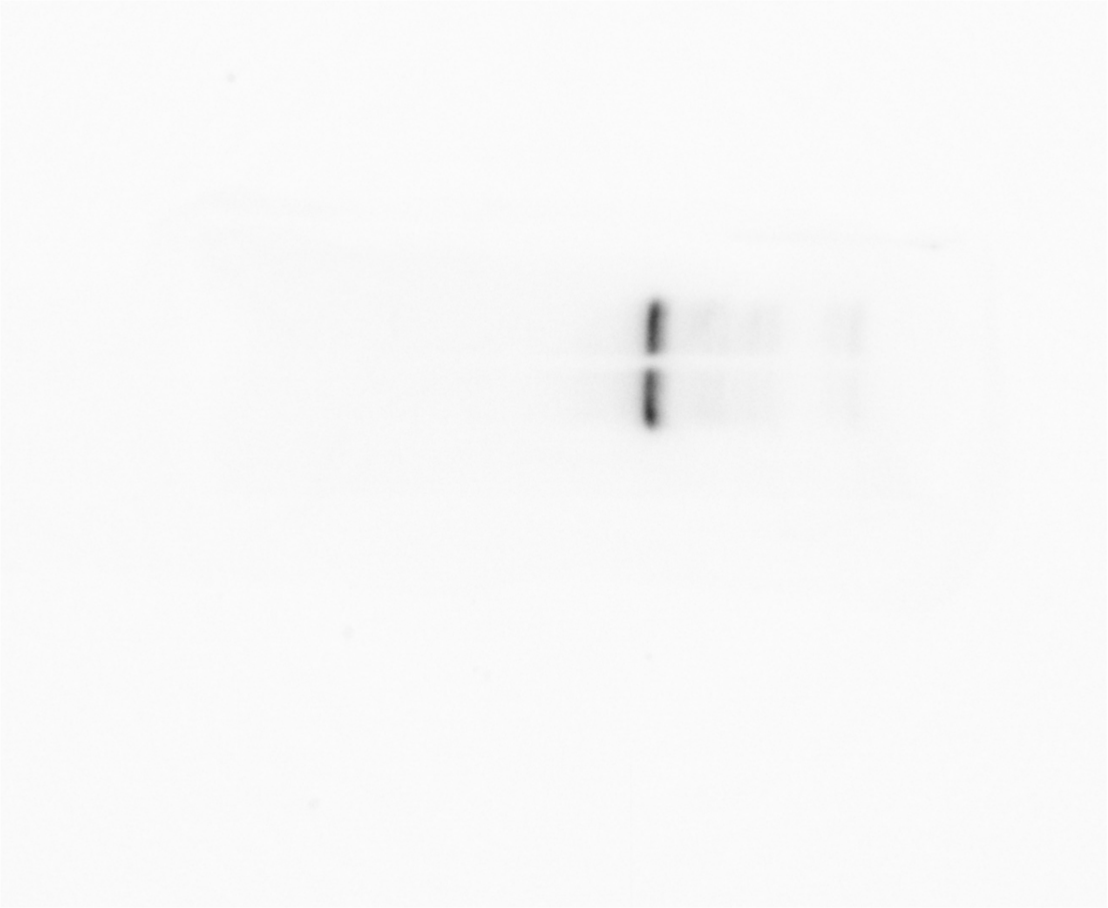

Original Image for Figure 2I_CCNE

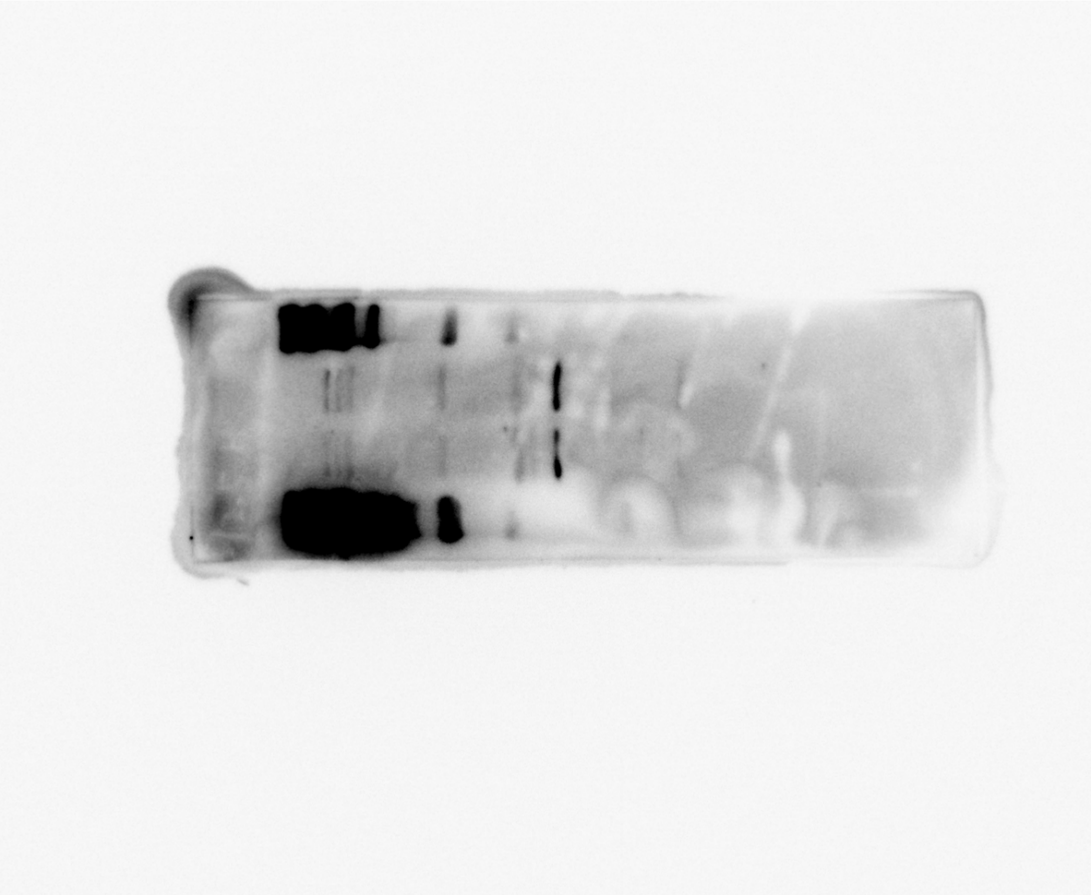

Original Image for Figure 2I_GAPDH

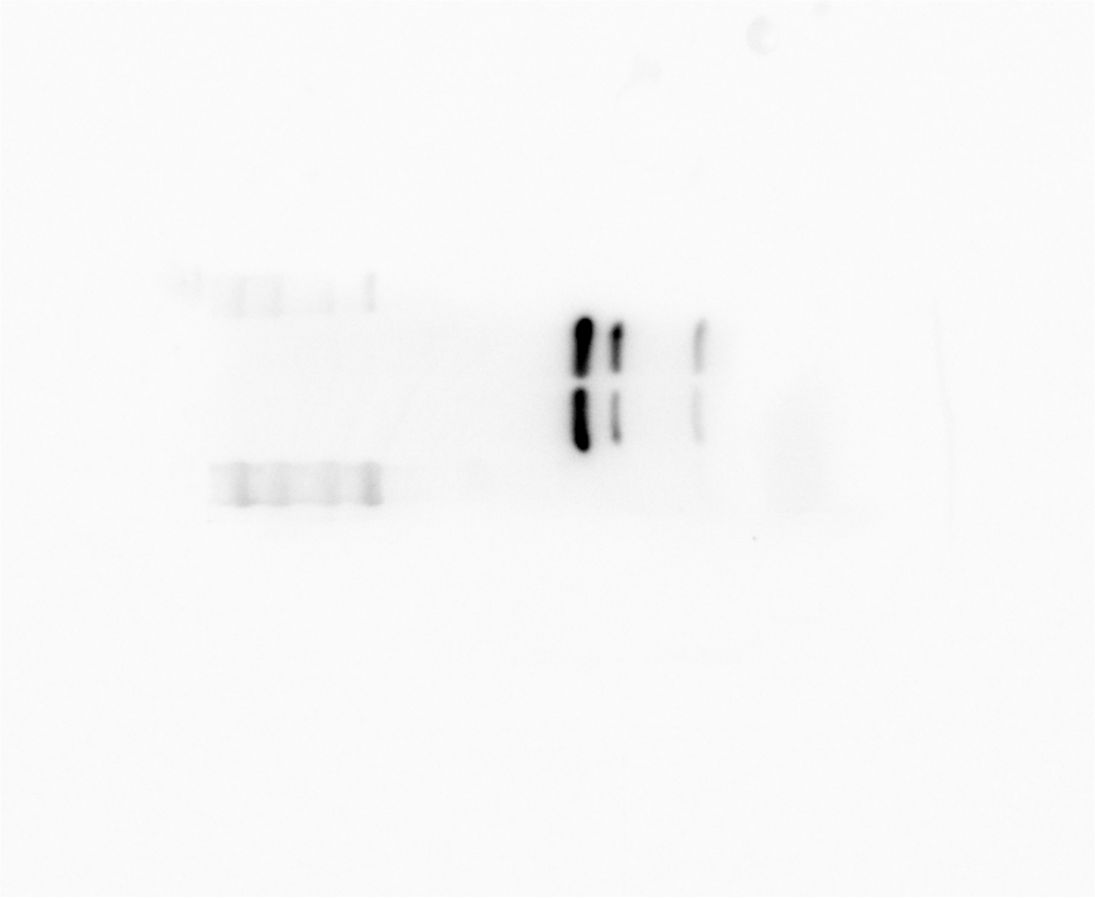

Original Image for Figure 2I_HSP90

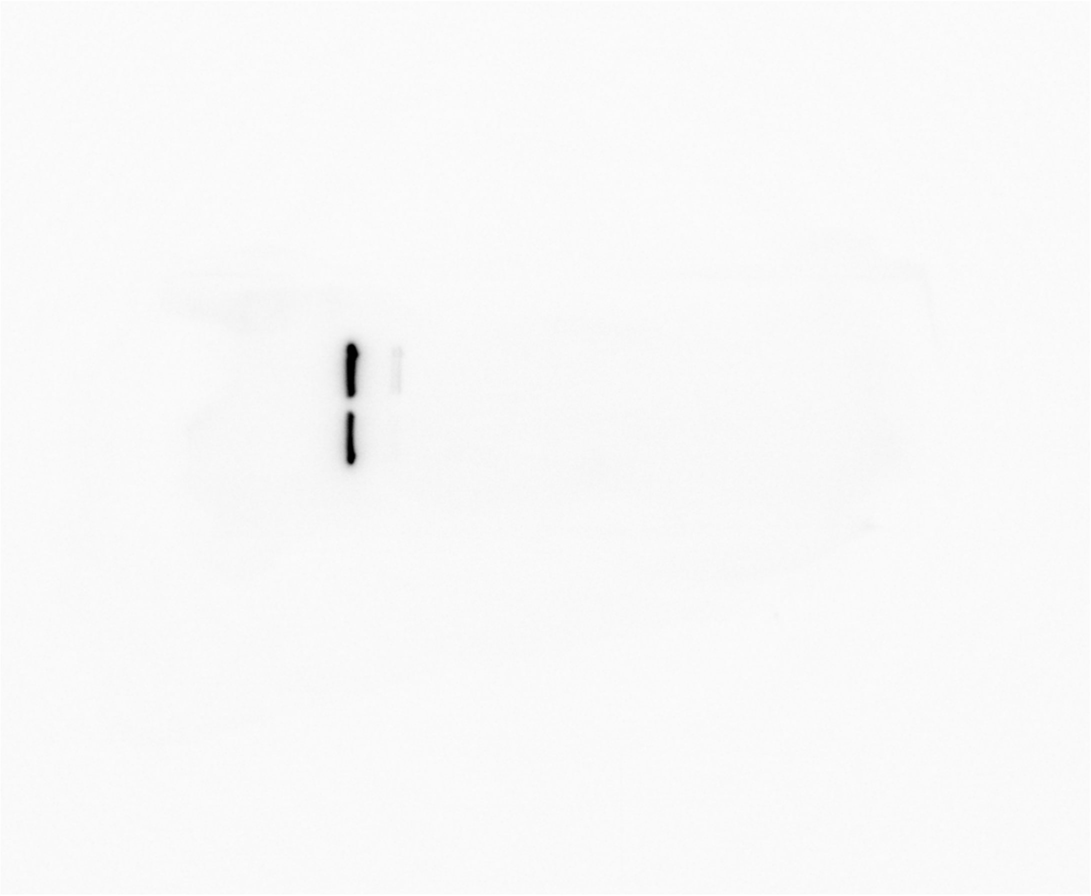

Original Image for Figure 2N_CAS3

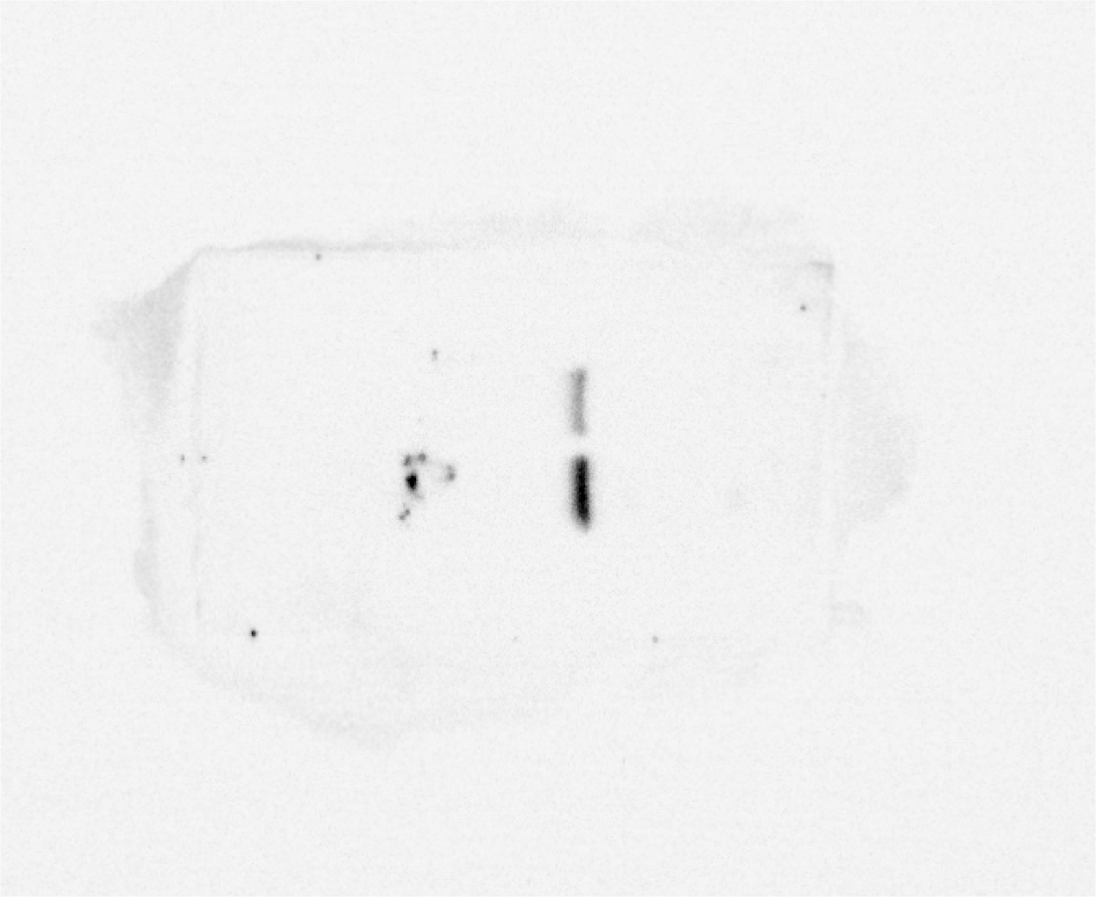

Original Image for Figure 2N_CLEAVED_CAS3

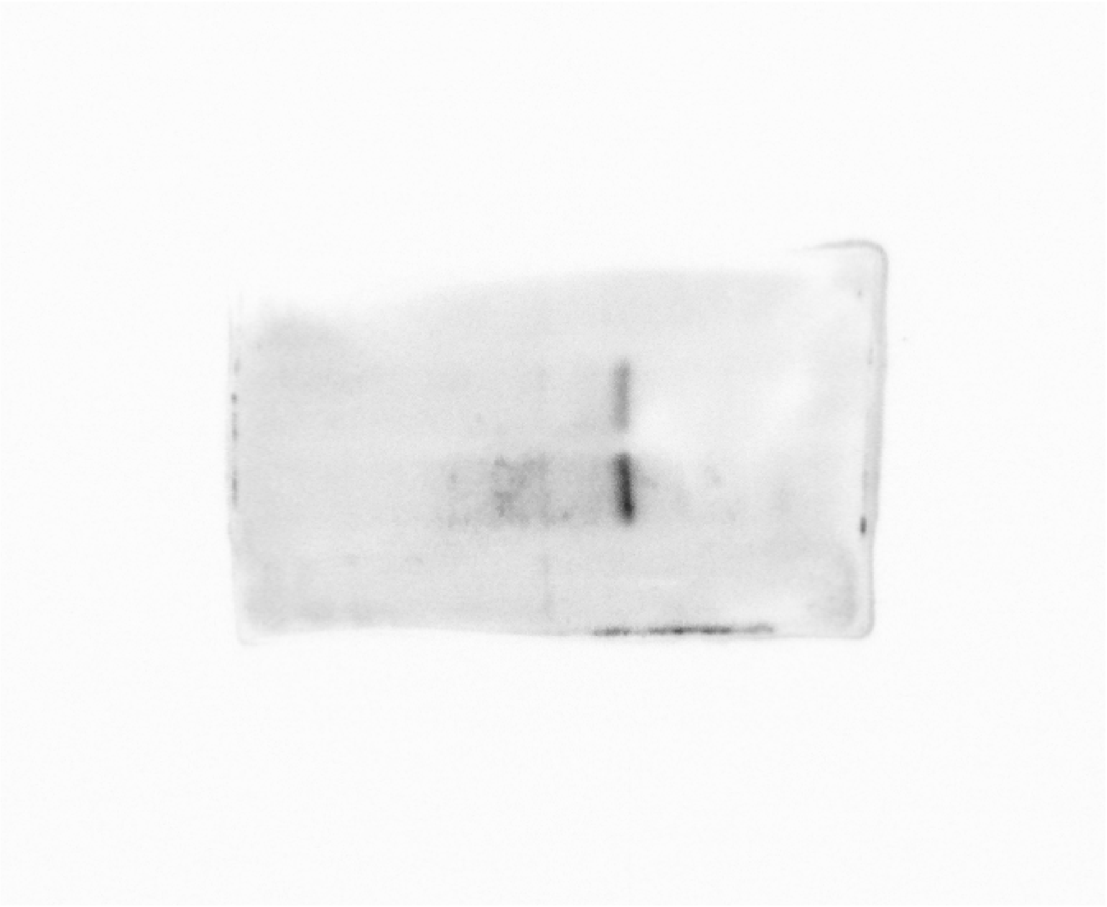

Original Image for Figure 2N_CAS7

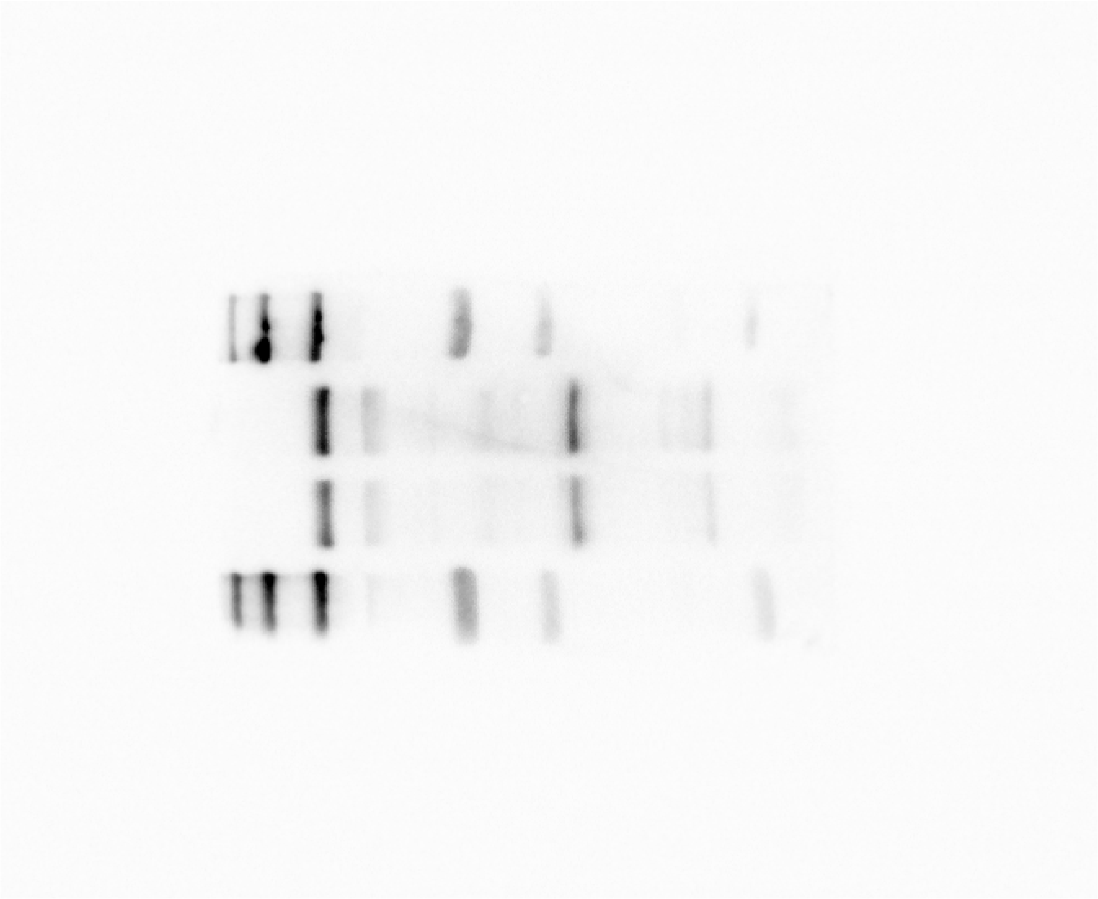

Original Image for Figure 2N_CLEAVED_CAS7

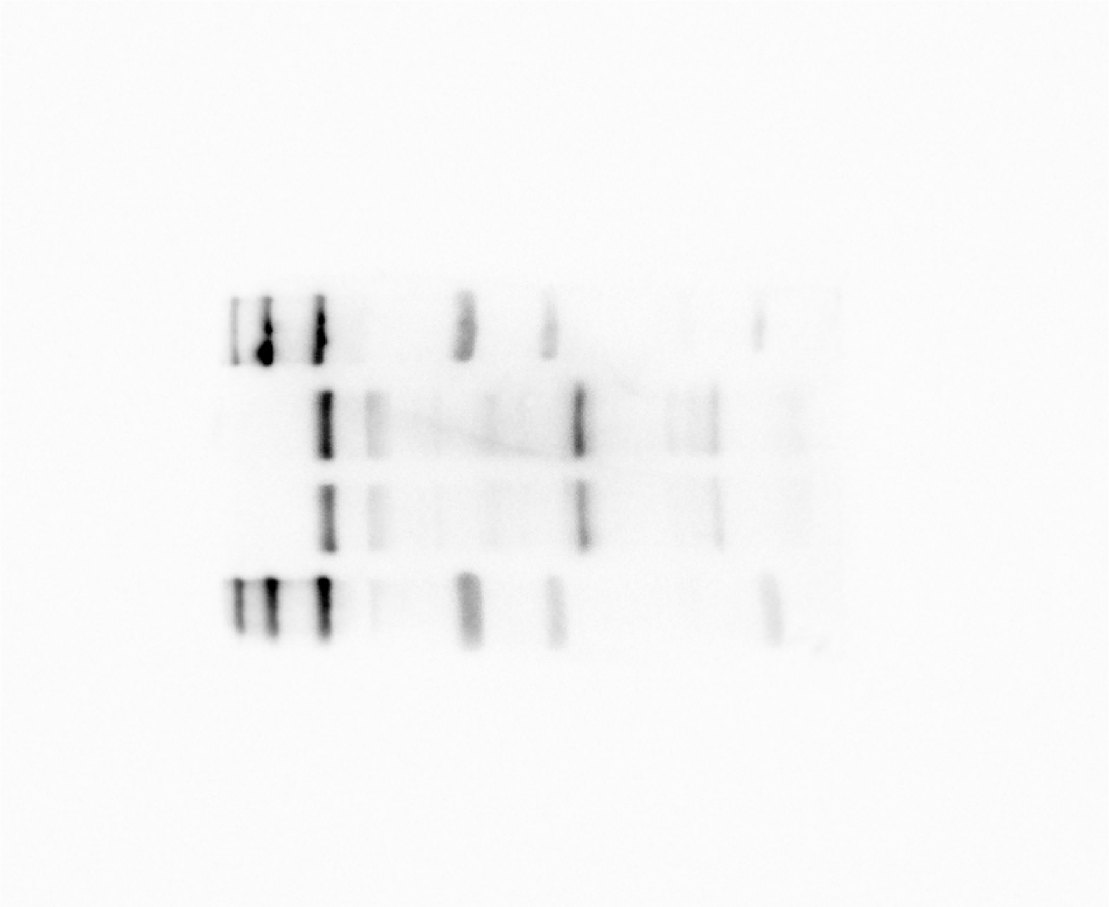

Original Image for Figure 2N_CAS9

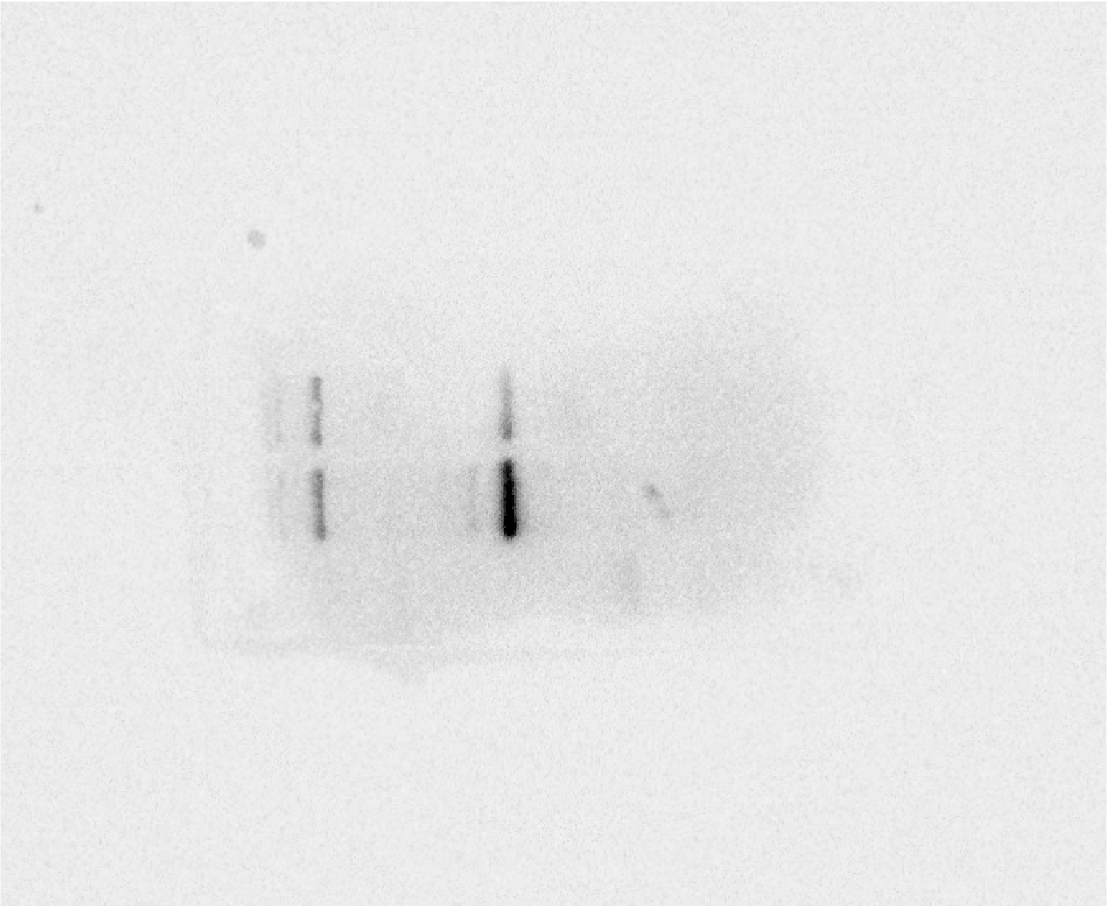

Original Image for Figure 2N_HSP90

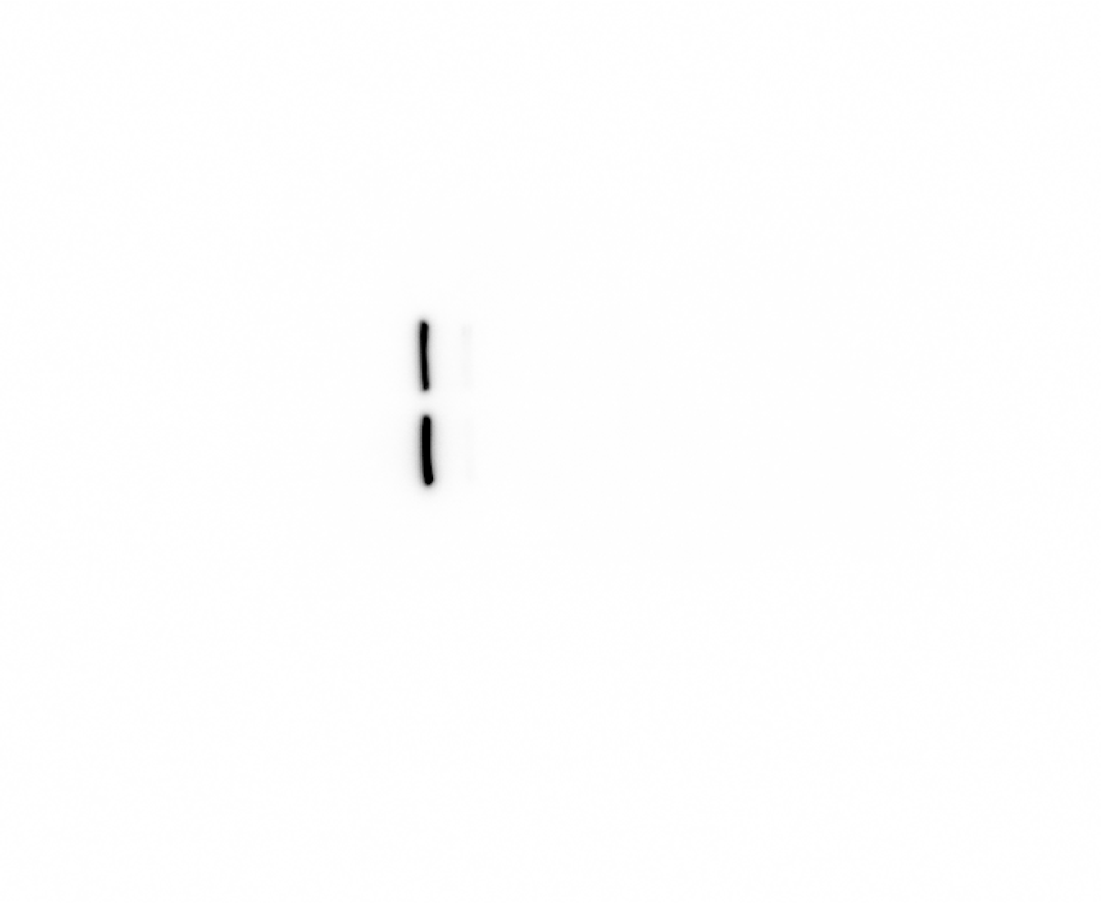

Original Image for Figure 4H_PI3K

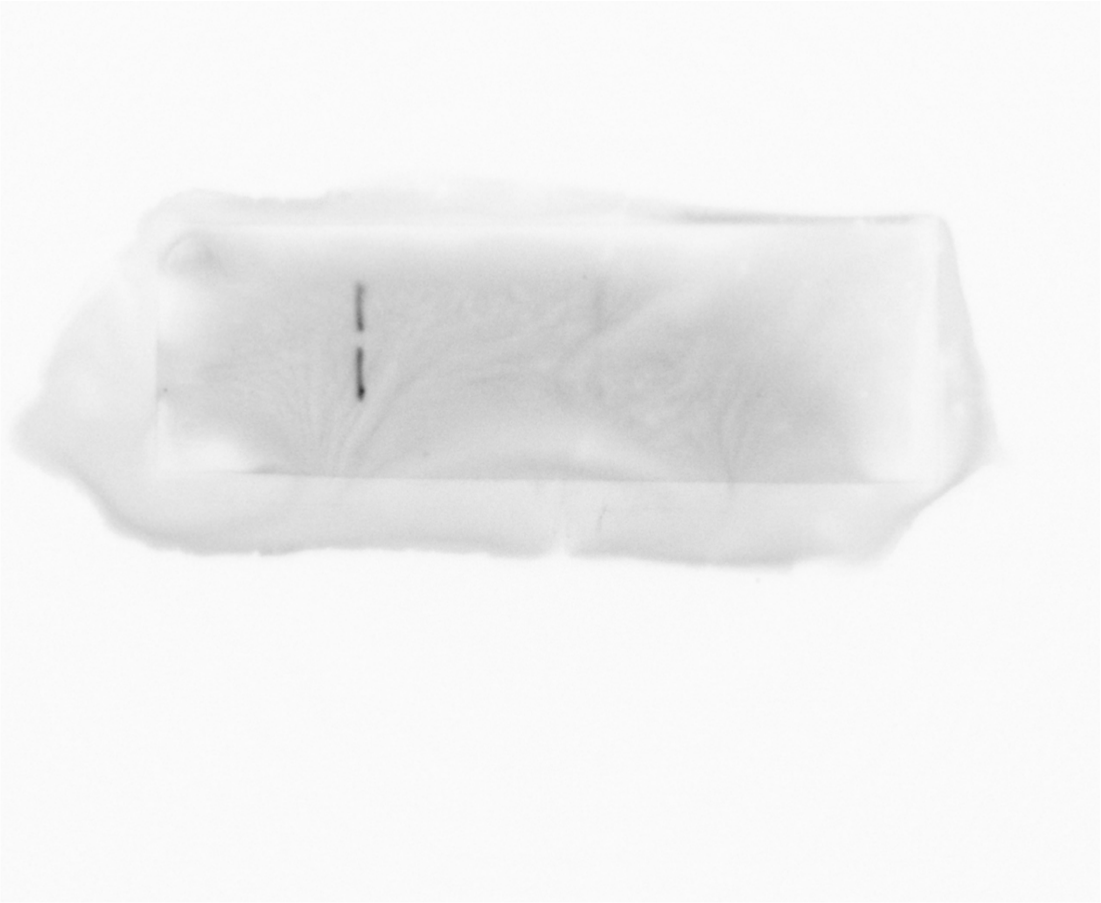

Original Image for Figure 4H_MTOR

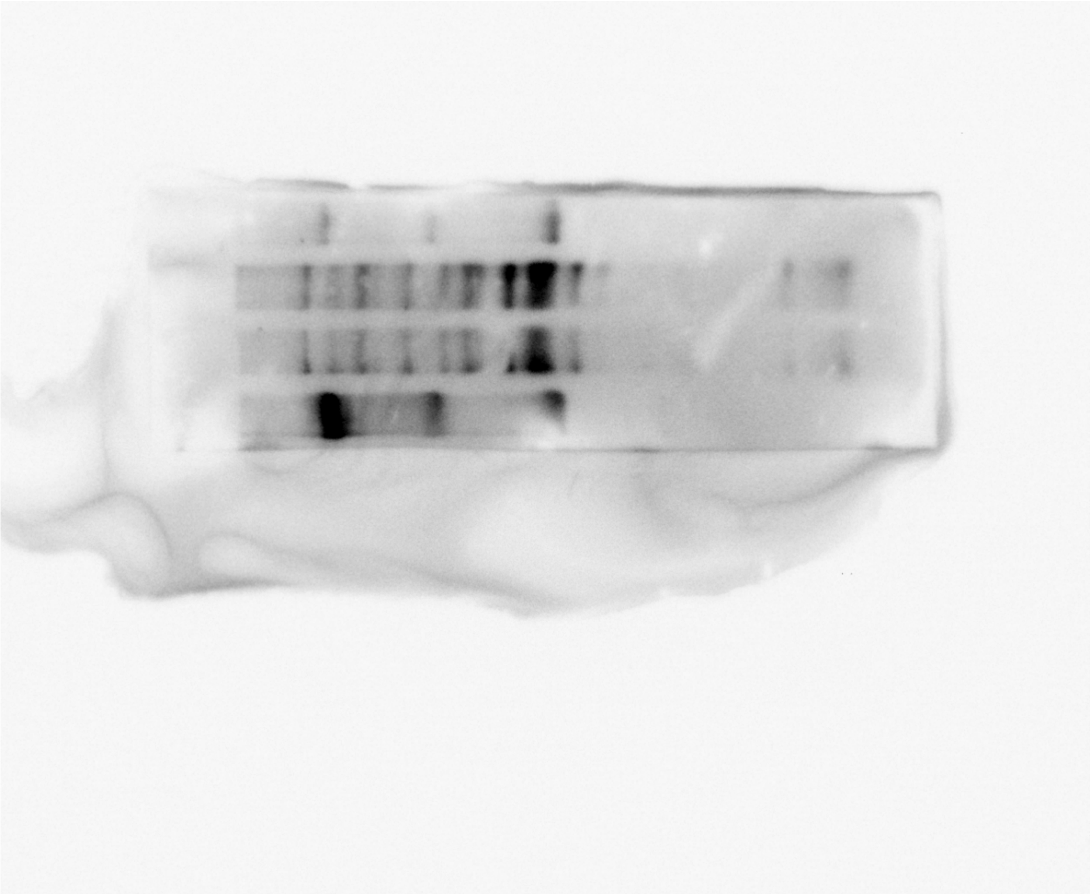

Original Image for Figure 4H_NFKB

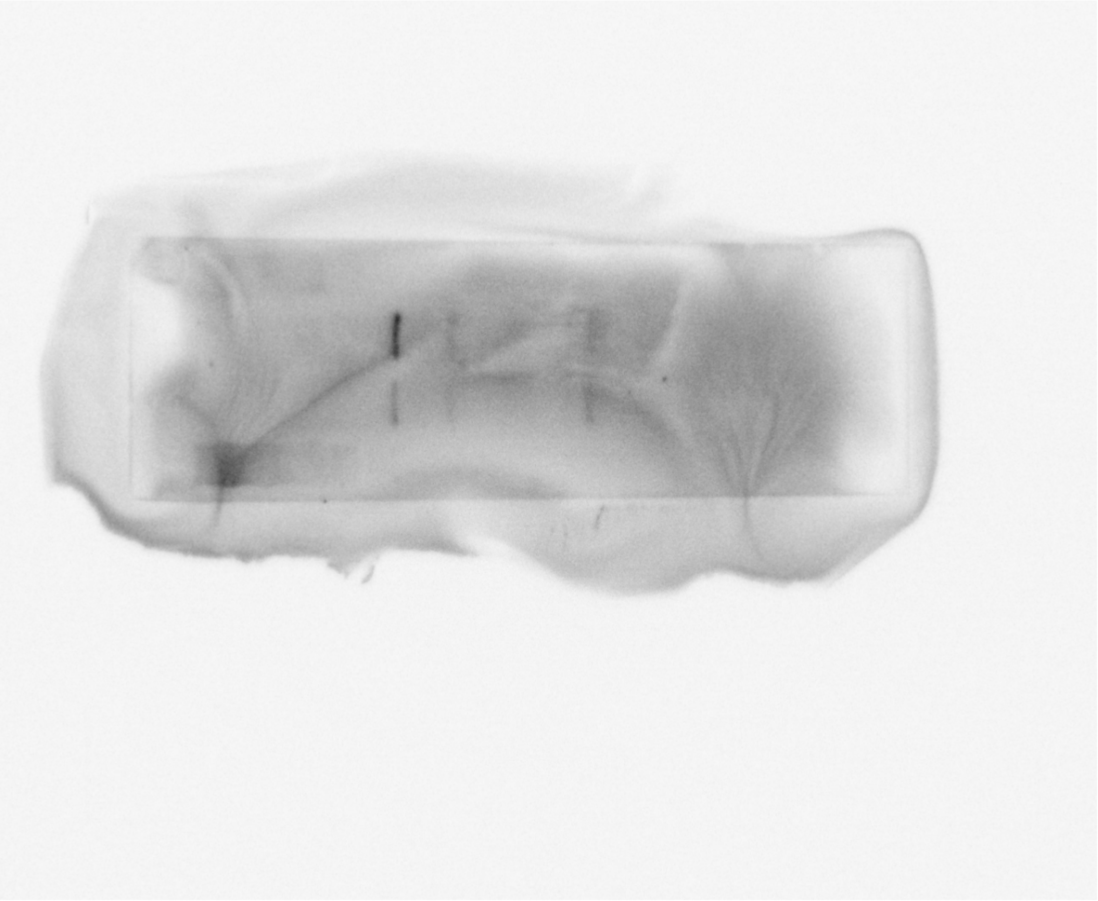

Original Image for Figure 4H_GAPDH

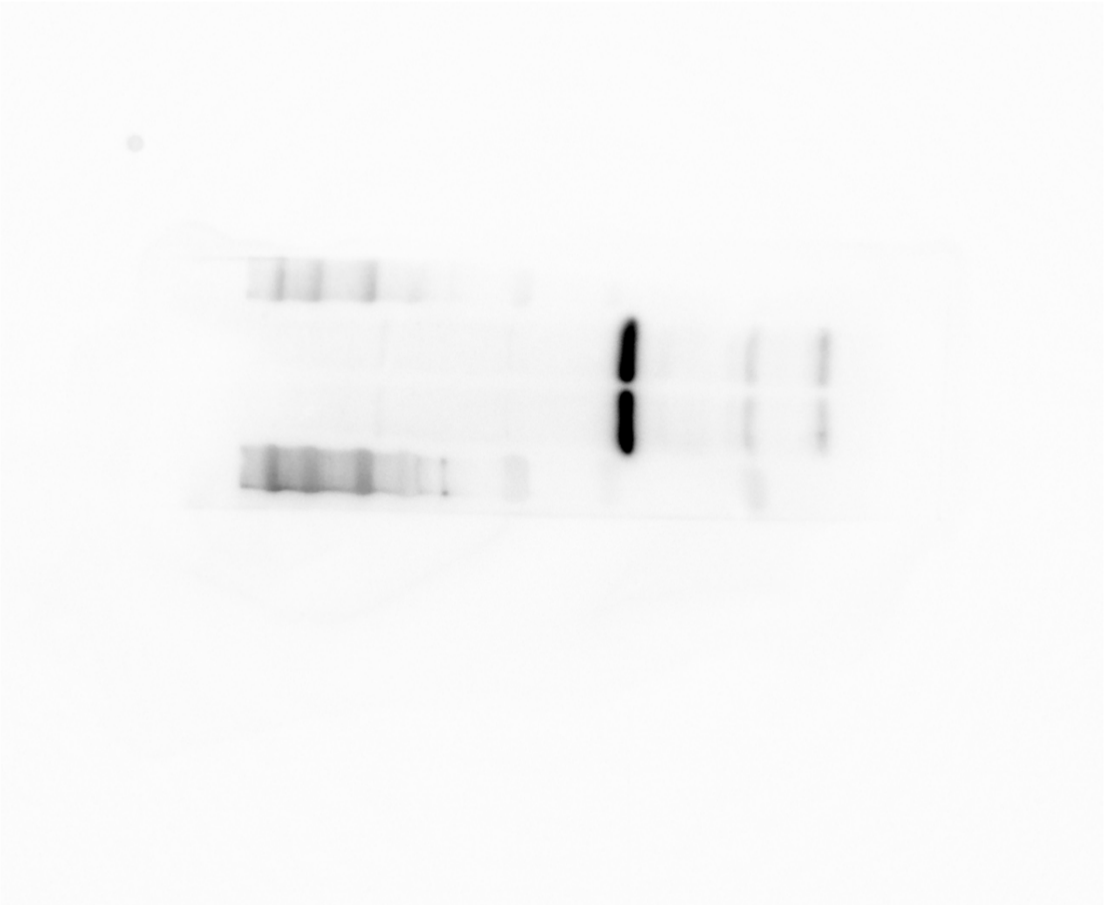

Original Image for Figure 4H_HSP90

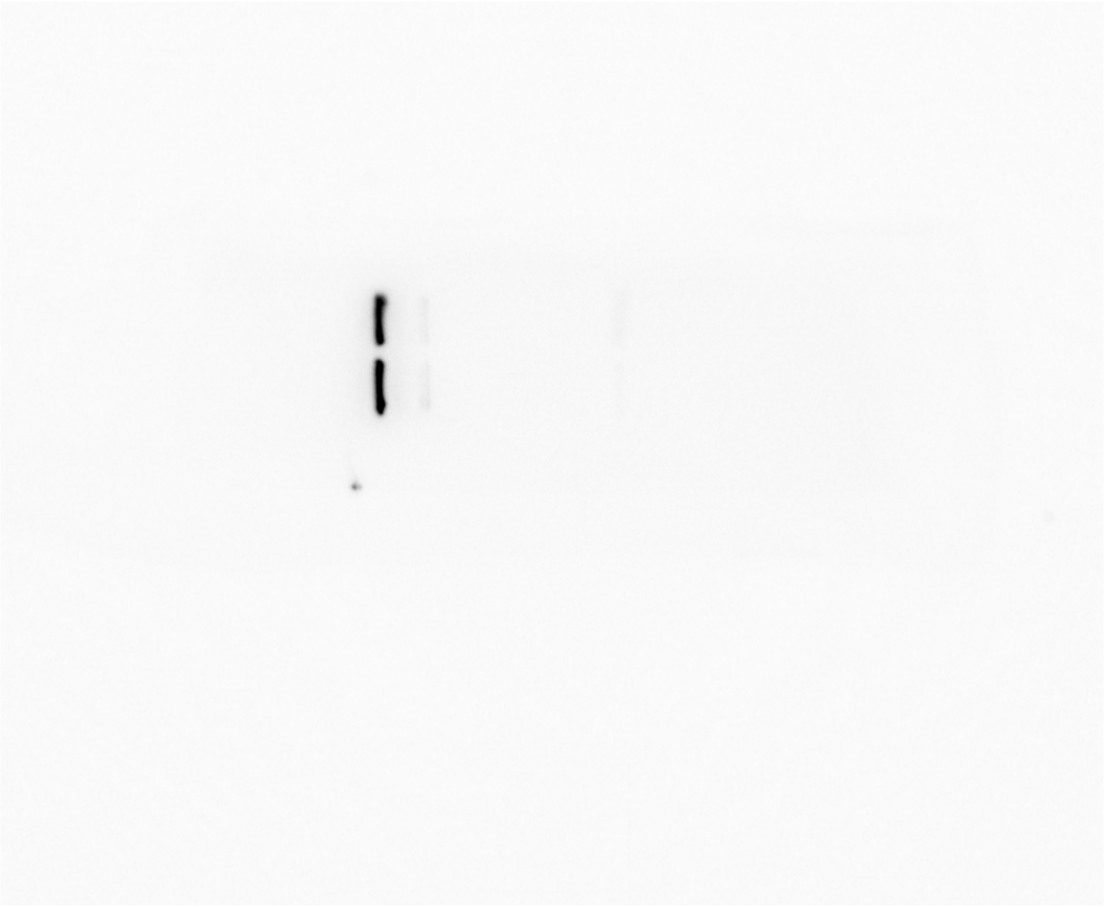

Original Image for Figure 5A

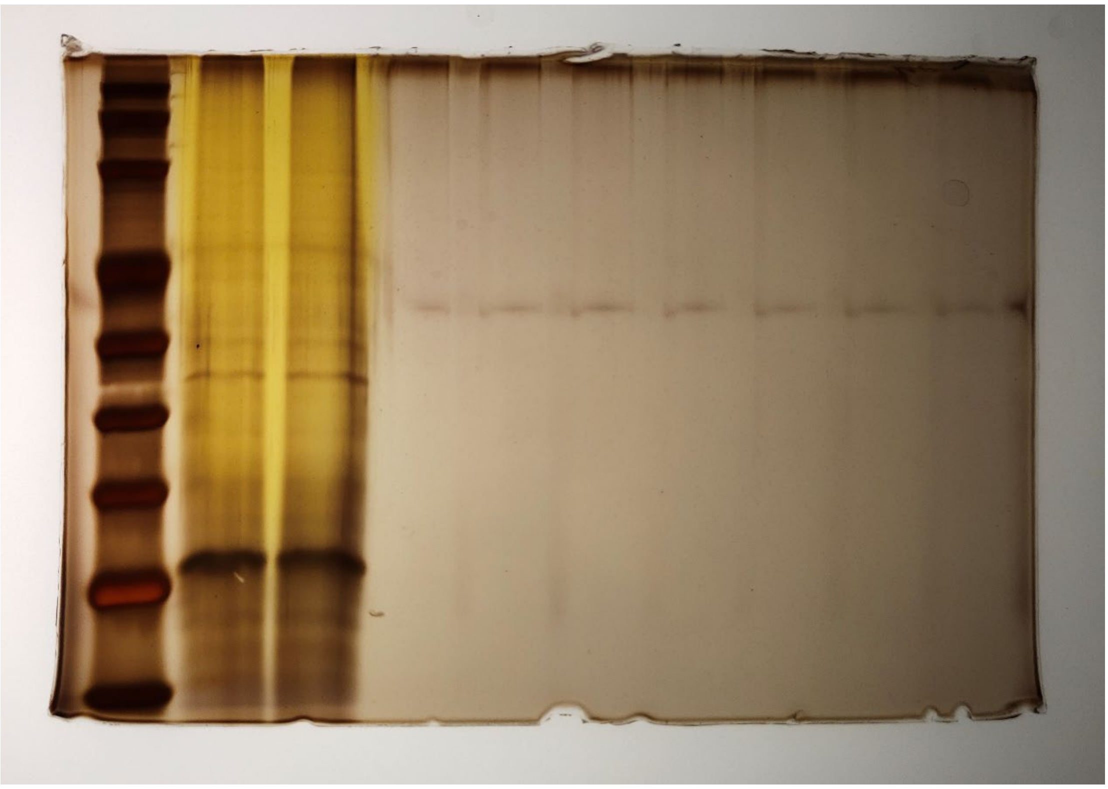

Supplement: Supplementary file 4 — Original Image for blots [file 41420_2023_1601_MOESM4_ESM.pdf]
